# Impact of rotating fan and ultrasonic humidifier on the thermal performance of pyramidal solar still

**DOI:** 10.1038/s41598-025-21863-0

**Published:** 2025-10-30

**Authors:** H. K. Sachidananda, Sampath Suranjan Salins, Kunal Ashok Mulchandani, Arpana S. Biradar, Shiva Kumar

**Affiliations:** 1 Manipal Academy of Higher Education, Dubai Campus, Dubai, 345050 PO UAE; 2 Department of Mechanical and Industrial Engineering, Manipal Institute of Technology, Manipal Academy of Higher Education, Manipal, 576104 India

**Keywords:** Pyramidal still, Ultrasonic humidifier, Evaporation, Still efficiency, Energy science and technology, Engineering

## Abstract

This study investigates the performance enhancement of a pyramidal solar still by integrating two active techniques: a low-power fan and an ultrasonic humidifier. Baseline experiments were first conducted using a static pyramidal solar still and compared with results from a fan-assisted configuration and a unit equipped with an ultrasonic humidifier. The fan promoted faster vapor circulation, while the humidifier produced fine mist particles that accelerated evaporation. Experiments carried out under Dubai’s climatic conditions showed that the system achieved a maximum efficiency of 57%, with corresponding values of heat transfer rate, evaporative heat transfer coefficient, and condensation heat transfer coefficient of 148.43 W, 20.38 W/m²°C, and 1.64 W/m²°C, respectively. The maximum partial vapor pressures at the basin and glass surfaces were 18,626.71 Pa and 15,276.53 Pa. At peak heating around 3:00 PM, the ultrasonic humidifier-based system recorded significantly higher partial vapor pressures—38.04% at the basin surface and 23.87% at the glass surface—compared to the static still, highlighting the effectiveness of active enhancements in accelerating vapor generation. Overall, the ultrasonic-based system demonstrated the highest performance, while the static unit produced the lowest output. The distillate yields were 90 ml, 180 ml, and 330 ml for the static, dynamic, and ultrasonic systems, respectively. Also, it is found that, the system yielded energy and exergy efficiencies equal to 25.11% and 1.75%.

## Introduction

 In the modern era, the availability of clean & safe drinking water remains a formidable challenge, especially in dry & semi-arid regions where freshwater is both scarce and frequently contaminated^1^. Leveraging the abundance of solar energy, solar distillation has gained prominence as an environmentally sustainable method for purifying saline or polluted water. A solar still emulates the natural hydrological cycle, employing solar radiation to induce evaporation, effectively separating water from dissolved salts, heavy metals, and pathogenic microorganisms^2^. The water vapor then condenses upon cooler surfaces, producing distilled water of high purity. The transparent cover of the still facilitates solar transmission while channeling the condensate to a designated collection area^3^. Conventional solar stills often experience low thermal efficiency and limited vapor condensation rates, challenges that are further aggravated by fluctuating environmental conditions. Therefore, modifying and enhancing solar designs is essential to maximize their output and overall performance^4^.

Active and passive solar stills primarily differ in their operational mechanisms and energy utilization. Passive solar stills depend exclusively on solar energy and natural convection offering a simple, low-cost solution with limited productivity. In contrast, active solar stills employ auxiliary energy sources or mechanical enhancements—such as fans, pumps, or ultrasonic humidifiers—to significantly improve heat and mass transfer, thereby increasing thermal efficiency and distillate yield^5^. However, this performance gain comes with added complexity, higher initial investment, and increased maintenance demands. The efficacy of a solar still is governed by a confluence of intricately linked factors that collectively dictate its efficiency and the volume of distilled water produced. Paramount design elements—such as the basin’s surface area, depth of water layer, inclination angle of glass cover, & quality of insulation—are instrumental in optimizing solar heat absorption while curtailing thermal losses^6^. Furthermore, ambient environmental conditions, encompassing intensity of solar irradiance, surrounding temperature, wind velocity & atmospheric humidity, exert profound influence over the evaporation and condensation dynamics intrinsic to the distillation process^7^. Augmentations in operation, including the incorporation of wicks, nanofluids, dyes, or phase change materials, serve to improve heat transfer rates & extend productive duration. The integration of active components—such as fans, humidifiers, and auxiliary heaters—may further amplify mass and heat transfer, albeit at the expense of increased system complexity. Meticulous upkeep, entailing regular cleansing of the glass cover and ensuring airtight sealing, remains indispensable for preserving optimal functionality over prolonged periods. Collectively, these determinants culminate in defining thermal performance & freshwater output of solar still^8^.

Numerous researchers have investigated solar still technology, and the details of their contributions are outlined below. Shivamallaiah et al.^9^ performed an empirical investigation on enhancing performance of a solar still by employing cooling of glass cover using cold water produced through an evaporative cooling technique. The chilled water, obtained by enveloping a water tank with a wet cotton wick, was sprayed over glass cover to augment the condensation process. The results demonstrated a 20.8% rise in condensation heat transfer coefficient & 7.3% improvement in overall efficiency. Additionally, cold water temperature was observed to be 20.4% lower than the ambient air temperature, thereby significantly improving the thermal performance of solar still. Sellami et al.^10^ sought to improve the productivity of solar still by incorporating an internal heat storage unit using blackened sponge layers. The findings revealed that a sponge thickness of 0.5 cm led to a 58% rise in distillate yield compared to baseline configuration. Panchal and Mohan^11^ aimed to enhance the solar still performance through the combination of fins, energy storage systems, and multi-basin configurations. Their findings revealed that fins effectively improved water distribution by increasing the basin’s surface area, thereby augmenting distillate output. Energy storage materials captured and retained thermal energy during sunshine time, subsequently liberating it during off-sunshine time to sustain productivity. Additionally, the multi-basin design utilized latent heat of condensation from lower basins to elevate the temperature of upper basin, further enhancing yield. The combined application of these techniques significantly improved water production efficiency. Jani and Modi^12^ conducted a evaluation of performance of a double slope single solar basin still integrated with circular & square hollow fins. The fins, fabricated from mild steel, were welded onto separate absorber plates, and tests were conducted under varying depths of the water basin. The findings revealed that a water depth of 10 mm resulted in highest output. Notably, solar still equipped with circular fins achieved a distillate yield of 1.49 kg/m²-day, while one with square fins produced 0.96 kg/m²-day. El-Samadony et al.^13^ conducted experimental investigation on stepped solar still combined with internal & external reflectors, an external condenser, and a suction fan. When compared to conventional solar still, the modified system gave a 66% increase in productivity with the condenser alone, while the combined use of reflectors and a condenser resulted in a substantial 165% improvement in distillate output. Mu et al.^14^ employed a refraction-based approach utilizing a Fresnel lens (FRL) to improve the productivity of conventional single-slope solar still. The FRL focused incident solar radiation onto basin bottom, thereby increasing high hourly productivity window (HHPW) and significantly improving heat transfer. The modified system gave a remarkable 467% rise in freshwater yield & achieved an overall efficiency of 84.7%. Yousef and Hassan^15^ examined the performance of a solar still integrated with various phase change material (PCM)-based thermal storage configurations to enhance freshwater productivity. Five configurations were evaluated: a conventional still, still with PCM, PCM with embedded pin fins, PCM with steel wool fibers, & steel wool fibers alone. Under identical climatic conditions, results revealed that the configuration incorporating PCM with embedded pin fins delivered the highest thermal performance and maximum distillate yield. Abu-Arabi et al.^16^ developed a solar still integrated with an external solar collector & phase change material (PCM), which effectively provided thermal energy during nighttime, thereby enhancing overall distillate yield. The study further revealed that reducing the overall heat transfer coefficient and increasing cooling water flow rate over the glass cover significantly improved system’s productivity. Winston et al.^17^ designed & evaluated a hybrid photovoltaic/thermal (PV/T) active solar still combined with single-slope configuration, assessing its output at varying water depths of 0.05 m, 0.10 m & 0.15 m. The system incorporated a solar-powered nickel-chromium (NiCr) heater and employed saline water to cool the PV module, thereby significantly enhancing both electrical efficiency and distillate yield. Experimental findings revealed that the hybrid still generated six times more freshwater and exhibited a 25% improvement in overall electrical and thermal efficiency compared to the old passive solar still. Manokar et al.^18^ integrated a solar still with photovoltaic (PV) panel to facilitate simultaneous desalination and power generation. The highest freshwater yield of 7.3 kg was achieved using inclined PV panels with both sidewall & bottom insulation, outperforming configurations with only sidewall insulation (4.4 kg) and without insulation (3.7 kg). This setup also demonstrated superior performance, attaining a solar still efficiency of 71.2% and exergy efficiency of 4.5%. Mevada et al.^19^ investigated the use of different energy storage materials (ESM) to improve performance of solar still. Materials with strong heat absorption and transfer properties—black glass balls, black granite & white marble—were tested in equal quantities. The solar still integrated with ESM achieved a daily yield of 2.5 kg/m², significantly outperforming the conventional design. The system also demonstrated an exergy efficiency of 12.55% and a solar still efficiency of 72.6%. Feria-Díaz et al.^20^ performed experimental investigations on single-slope solar still, assessing its performance under varying climatic conditions. The study reported an average distilled water output of 1.57 L per square meter per day. Yadav and Prakash^21^ conducted an analysis of solar still fabricated from natural fiber reinforced composite material, incorporating transparent side walls & vertical basin design. Relative to conventional model, this enhanced configuration exhibited a notable 31.52% augmentation in distillate yield. Senthilkumar et al.^22^ conducted a performance evaluation of a pyramid solar still (PSS) enhanced with different energy storage materials, namely Kanche marbles & natural banana fibers, in comparison to a baseline unit employing only black paint on the basin. The findings revealed that the configurations utilizing Kanche marbles and banana fibers yielded substantially higher distillate outputs of 2.94 kg and 2.85 kg, respectively, thereby significantly surpassing the performance of the conventional setup. Mohammed et al.^23^ conducted a comparative study between a conventional pyramid solar still (CPSS) & modified pyramid solar still (MPSS) incorporating a PV panel, pump, spiral coil, and Arduino Mega unit. The MPSS exhibited a remarkable 131% rise in daily freshwater output over CPSS & achieved a significantly higher energy efficiency of 56%, compared to 24% for the conventional design. Eswaran et al.^24^ investigated the output performance of solar still combined with evacuated tube collectors, enhanced by the incorporation of nanoparticles like Copper oxide (CuO), Aluminium oxide (Al₂O₃), & Zinc oxide (ZnO) to improve thermal conductivity & heat generation. The inclusion of these nanoparticles led to notable increases in performance—17.4% for CuO, 15.7% for Al₂O₃, and 14.5% for ZnO, respectively—demonstrating their effectiveness in augmenting the efficiency of system. Abdallah and Aldarabseh et al.^25^ investigated a conical solar still design with varied volume flow rates, achieving a remarkable solar still efficiency of 69%. Essa et al.^34^ worked on a concept which improved the pyramid solar still performance by integrating a triangular vertical absorber with single-axis tracking, burlap-covered flat/corrugated surfaces to boost evaporation, a rear mirror for enhanced solar capture, a fan for faster steam removal, and a phase changing material–silver nanoparticle layer for better thermal performance. Compared to the reference still, productivity increased by 32% with the triangular absorber, 61% with the corrugated triangular absorber, and 102% with the corrugated triangular absorber plus tracking. Essa et al.^35^ developed a novel hemispherical solar distiller (HSD) incorporating jute wicks and thermal storage materials (paraffin wax combined with graphite and aluminum oxide nanoparticles) to enhance potable water production in remote regions. With a projected area of 5000 cm², the HSD achieved freshwater yield improvements of 41.73% without TSM and 90.74% with TSM compared to a conventional solar distiller (CSD). Further integration of a 20 W electric heater and an external condenser boosted productivity by 145% over CSD. Overall, the HSD demonstrated superior performance in both yield and efficiency, producing 6370 mL/m²/day with energy and exergy efficiencies of 48.2% and 4.9%, respectively. El-Sebaey et al.^36^ investigated a semi-cylindrical stepped-basin solar still (SCSBSS) integrated with paraffin wax (PCM) to improve thermal performance. Compared to a conventional semi-cylindrical solar still (SCSS), the PCM-based system achieved 44.52% higher productivity, yielding 3889 mL/m²·day at 20 L saltwater depth under Egyptian conditions. Economic analysis showed a cost per liter of 0.0157 US$/L and a payback period of 123 days over a 10-year lifespan. The thermal and exergy efficiencies were 36.86% and 3.27%, respectively. Sathyamurthy et al.^37^ addressed the freshwater scarcity by enhancing a single slope solar still (SSSS) with paraffin wax stored in recycled soda cans. The cans were coated with black paint and carbon soot nanoparticles (50–60 nm) from engine exhausts to improve heat absorption. Tests compared three setups: SSSS without PCM, with uncoated PCM cans, and with coated PCM cans. The coated PCM system achieved 75.7% higher thermal performance and 102.3% greater yield than the other configurations. The approach highlights a sustainable method combining waste recycling, energy storage, and renewable desalination. Rajamony et al.^38^ generated a binary nanocomposite of silver (Ag) and coconut shell biochar (CSB) which enhanced A46 PCM via a two-step method which was used in the solar stills. It achieved a 102.15% rise in thermal conductivity (0.23 → 0.465 W/m·K), increased energy storage (158.6 → 167.8 J/g), and 2.05× higher optical absorbance. The material remained stable after 500 cycles and improved thermoelectric generator voltage by 7.98%, showing strong potential for thermal energy storage in the 44–48 °C range. Mousa et al.^39^ developed a modified solar distiller with a flat-plate collector and gravity-assisted heat pipes was developed at Menoufia University, Egypt. Tests compared two conventional stills (2 cm and 9 cm basin depths) with the modified design (9 cm depth). Daily productivities were 1.49, 1.67, and 1.75 L/m², respectively, giving 12.55% and 17.93% improvements over the 2 cm conventional still. CFD modeling showed good agreement, with average deviations of 11.74% for absorber temperature, 7.53% for glass cover temperature, and 9–30.9% for productivity. S. El-Sebaey et al.^40^ developed a 3D multi-phase CFD model to predict solar still performance without experimental input, using a solar radiation model. Results showed good agreement with experiments: daily productivity was 1.98 L/m² (simulated) vs. 1.79 L/m² (measured) at 2 cm depth, with efficiencies of 16.79% and 15.5%, respectively. El-Sebaey et al.^41^ designed, fabricated, and tested a single-slope double-basin solar still (SSDBSS) as a renewable desalination system. Compared with a conventional single basin still (SSSS), the SSDBSS achieved 59.9% higher productivity (2.86 vs. 1.79 L/m²·day) and 61.3% greater thermal efficiency at 2 cm water depth. Mevada et al.^42^ enhanced solar still (SS) performance using a zig-zag air-cooled condenser (ZZACC) and CuO nanomaterial. Compared to a conventional still (CSS), the modified system (ZZACC with CuO) achieved 46.8% higher distillate output and 46% higher energy efficiency, along with improved exergy and latent heat performance due to increased evaporative heat transfer. Essa et al.^43^ optimized the rotating spherical solar distiller (RBSD) with wick, preheater, and condenser to boost productivity. The best speeds were found to be 0.5 rpm with wick and 1 rpm without wick, increasing yield by 62% and 45%, respectively. Preheating at 65 °C raised output to 6200 mL/m²·day, a 91% gain over the conventional still CSD which yielded 3250 mL/m²·day.

Following an extensive literature review, numerous researchers have explored both active and passive solar stills, employing a variety of techniques to enhance performance. These include glass cover cooling via evaporatively cooled water, internal heat storage using blackened sponge layers, integration of circular and square hollow fins, advanced energy storage systems, multi-basin configurations, internal and external reflectors, external condensers, suction fans, Fresnel lens (FRL) utilization, phase change materials (PCM), external solar collectors, photovoltaic (PV) panels, and the incorporation of materials such as black glass balls, black granite & white marble. Additionally, innovations involve the use of pumps, spiral coils, Arduino Mega units, and nanoparticles including CuO, Al₂O₃, and ZnO to further improve solar still efficiency.

Although extensive research has been conducted on single- and multi-basin solar stills, studies specifically addressing pyramidal solar stills remain limited. In addition, literature exploring dynamic (fan-assisted) and ultrasonic solar stills—especially in direct comparison to conventional static systems—is scarce. Likewise, comparative assessments of thermal and distillation performance across static, dynamic, and ultrasonic configurations have not been comprehensively investigated. To address these gaps, an experimental pyramidal solar still was designed and fabricated, integrating a rotating fan to enhance forced convection and an ultrasonic humidifier to improve vapor generation. Experiments were performed between 11:00 AM and 4:00 PM, during which key thermal parameters were systematically recorded for each configuration. The performance of the static, dynamic, and ultrasonic systems was rigorously evaluated, and a detailed comparative analysis was carried out to assess the effectiveness of passive and active enhancement techniques.

### Novelty of the study

1. Incorporating active enhancement techniques—such as ultrasonic humidification and dynamic airflow generated by rotating fans—within the pyramidal structure constitutes an innovative strategy to markedly enhance heat and mass transfer rates.
2. Comparative evaluations of thermal performance among static, dynamic, and ultrasonic solar stills in a pyramidical solar still.

## Methodology involved and mathematical expressions

**Fig. 1:**
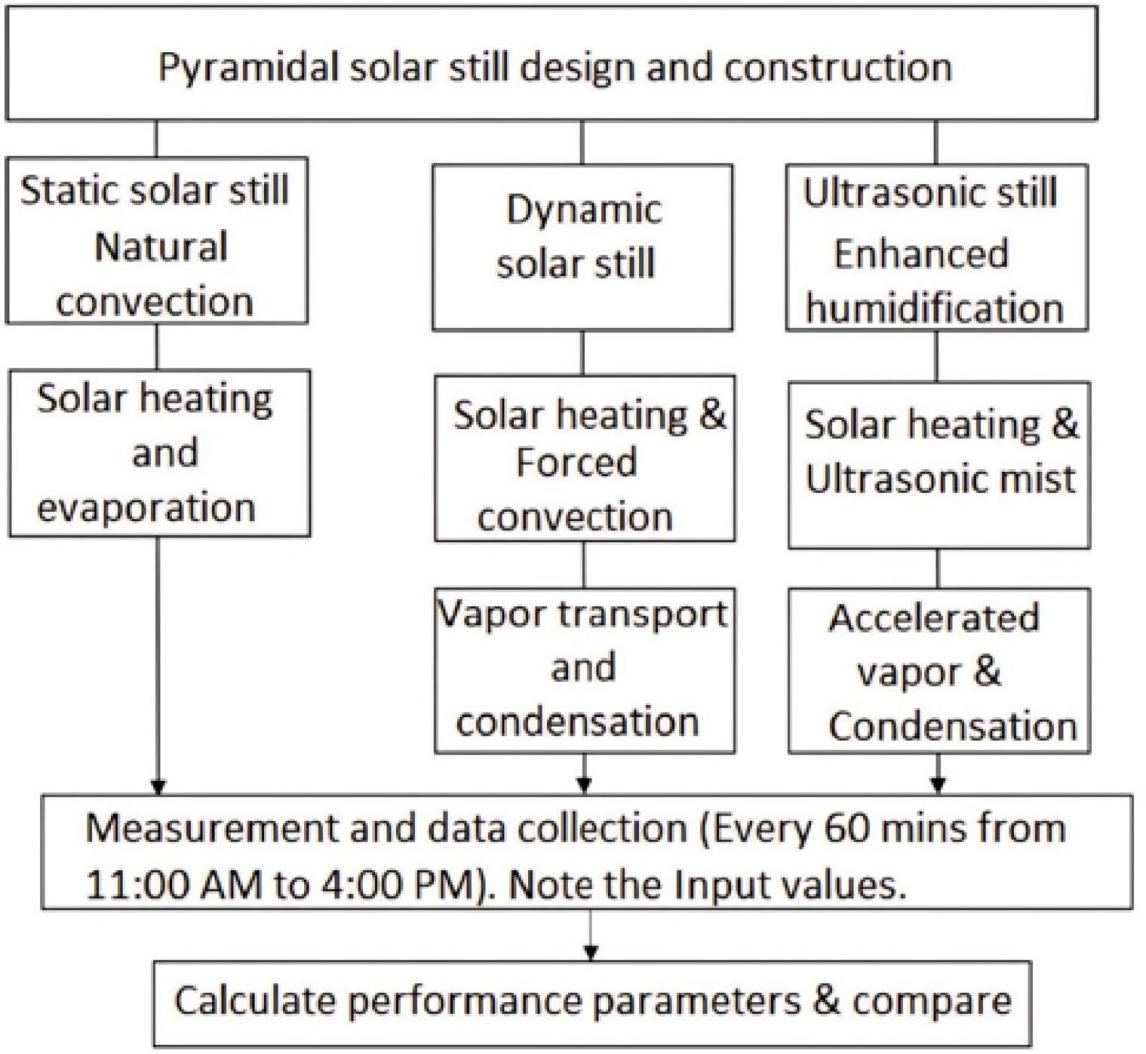
Methodology of the pyramidal solar still experiment.

Figure 1 illustrates the experimental methodology employed in the study of the pyramidal solar still. In this investigation, three distinct configurations of a pyramidal solar still—namely static, dynamic (rotating fan-assisted), and ultrasonic humidifier-enhanced—were experimentally assessed. Each unit was constructed with transparent pyramidal glass cover & black-coated basin to optimize solar energy absorption. The static configuration relied solely on natural convection without external intervention. In contrast, the dynamic setup incorporated a rotating fan to induce forced convection, thereby accelerating vapor movement and improving both heat and mass transfer within the chamber. The ultrasonic variant employed an ultrasonic humidifier to produce fine water vapor mist, substantially increasing the rate of humidification and subsequent condensation. All systems were evaluated under uniform outdoor climatic conditions between 11:00 AM and 4:00 PM, with temperature readings of the basin, glass cover & water, along with distillate yield, recorded at hourly intervals. The resulting thermal performance and freshwater output were critically analyzed to quantify the performance enhancements achieved by the dynamic and ultrasonic configurations over the baseline static model. The performance parameters related to the pyramidal solar still experiment are determined using following expressions.

The evaporative heat transfer coefficient (h_ew_) represents rate of thermal energy transfer from basin water to glass cover because of evaporation within solar still. The evaporative heat transfer coefficient is given by Eq. (1) where h_ew_ is the evaporative heat transfer coefficient, W/m^2^⁰C, h_cw_ is a condensation heat transfer coefficient W/m^2^⁰C, P_w_ & P_g_ are saturated vapor pressure at water & glass temperature, Pa, T_W_ & T_g_ are the water & glass temperature, ⁰C^9,18^.

1 $$\:{h}_{ew}=16.273\:x\:{10}^{-3}x{h}_{cw}\left(\frac{{P}_{w}-{P}_{g}}{{T}_{w}-{T}_{g}}\right)$$

The condensation heat transfer coefficient is given by Eq. (2). The condensation heat transfer coefficient (h_c_​) quantifies the rate of latent heat liberated during phase change of water vapor into liquid on inner surface of glass cover in a solar still. This parameter significantly influences condensation rate and, consequently, the overall productivity of the system^9,10^.

2 $$\:{h}_{cw}=0.884\:x\:{\left[{T}_{w}-{T}_{g}+\frac{\left({P}_{w}-{P}_{g}\right)\left({T}_{w}-{T}_{g}\right)}{268.9\times\:{10}^{3}-\:{P}_{w}}\right]}^{1/3}$$

The partial vapor pressure at the basin surface (P_W_), Pa, corresponds to saturated vapor pressure at water temperature, indicating the potential for evaporation. Conversely, the partial vapor pressure at glass cover (P_g_),Pa, is dictated by the glass surface temperature and governs the condensation process. The difference between these two pressures serves as the driving force for phase change phenomena within the solar still.

Where $\:{P}_{w}$ and $\:{P}_{g}$ are given by the Eqs. (3) and (4)^9,18^.

3 $$\:{P}_{W}={e}^{\left(25.317+\frac{5144}{{T}_{W}+273}\right)}$$

4 $$\:{P}_{g}={e}^{\left(25.317+\frac{5144}{{T}_{g}+273}\right)}$$

The heat transfer rate, W/m^2^ in a solar still which is given by Eq. (5) is determined by combined contributions of evaporative, convective & radiative mechanisms operating between basin water and glass cover. This integrated thermal interaction governs efficiency of distillation process, with each mode playing a critical role in facilitating the phase transition of water^9,18^.

5 $${\dot{Q}}=h_{ew}\times(T_W-T_g)$$

The efficiency of solar still represents effectiveness with which available solar energy is converted into usable distilled water. It is given by ratio of heat transfer rate to incident radiation, W/m^2^^9,18^.

6 $$\:{\upeta\:}=\frac{\dot{Q}}{I\left(t\right)}$$

The Eq. (1) to (6) are used to determine the output parameters of solar still.

## Construction, working and instrumentation

### Construction

**Fig. 2:**
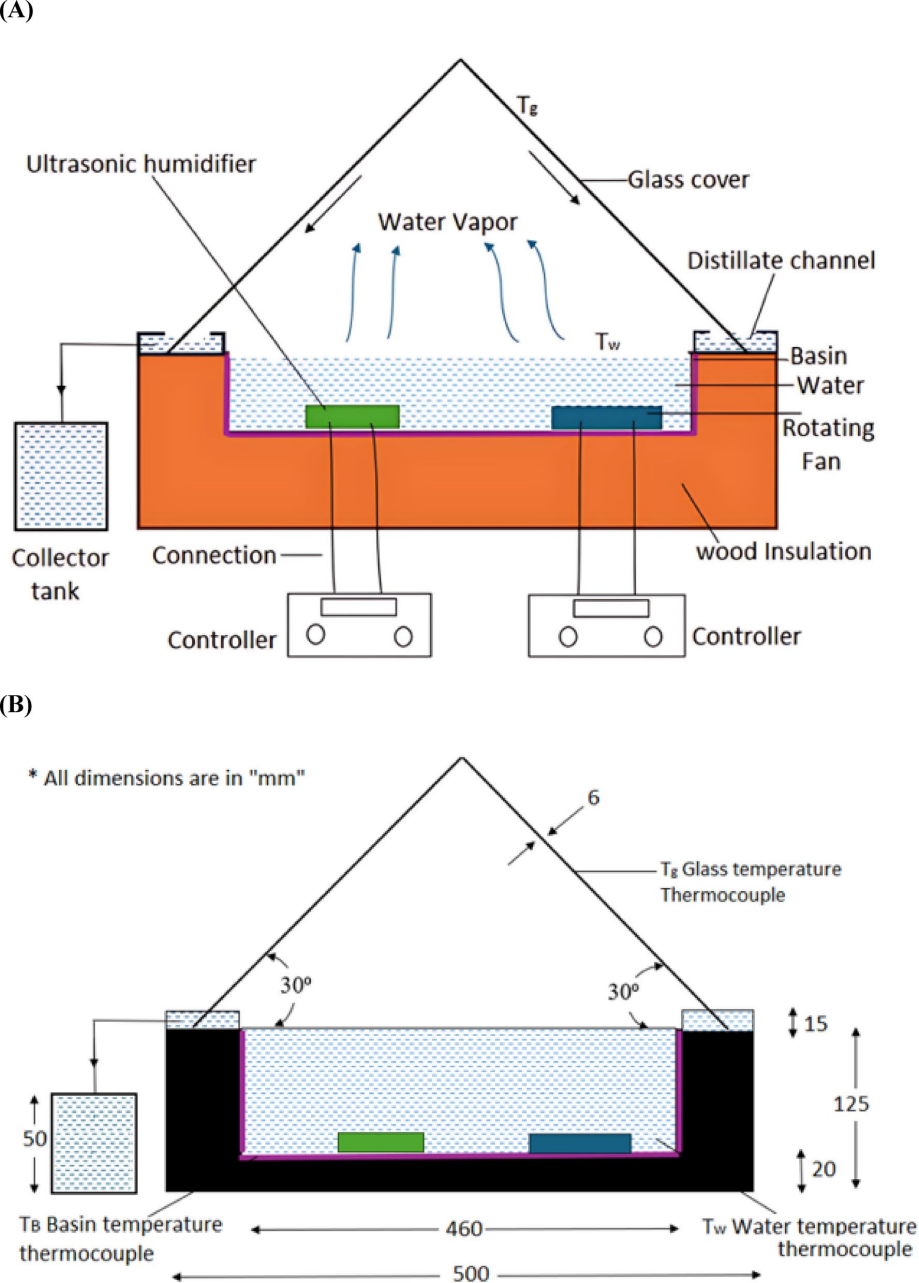
(**A**). Schematic sketch of pyramidal solar still. (**B**). Dimensions of the pyramidal solar still model with the thermocouple positions.

The comprehensive model of the pyramidal solar still is systematically delineated into three principal components. The first is the basin, serving as the foundational structure designed to contain saline or impure water designated for purification. Its shallow configuration facilitates accelerated evaporation, while thermal insulation mitigates heat dissipation. The square basin is made of four sheets of polished aluminum metal of 50 mm x 10 mm x 1 mm dimensions and the insulation is made of 50 mm x 20 mm x 10 mm wooden ply. Aluminum, being lightweight, corrosion-resistant, and highly conductive, efficiently transfers solar heat to water, accelerating evaporation. A black coating enhances its heat absorption and offers added oxidation protection. Wood, used for basin casing, provides effective insulation, retains heat, and supports energy efficiency while being easy to shape. Aluminum and wood were selected for the solar still basin due to their combined thermal efficiency, durability, and cost-effectiveness. Aluminum’s high thermal conductivity enhances evaporation, while its light weight and corrosion resistance make it practical and economical compared to copper. Wood provides structural support and thermal insulation, reducing heat losses and ensuring durability. Together, they offer an optimal balance of performance, ease of fabrication, and suitability for both experimental and larger-scale applications. The second component is the transparent pyramidal top cover—an elegantly crafted glass structure shaped to optimize solar radiation capture throughout daylight hours. The pyramidal top cover consists of four 6 mm tempered glass panels, designed to maximize solar gain and enhance condensation through a temperature gradient between the inner glass surface and ambient air. The durable, thermally resistant, and highly transparent glass creates a greenhouse effect, while its sloped design ensures efficient condensation and smooth drainage of distilled water. The third component encompasses the enhancement mechanisms, comprising two distinct systems meticulously engineered and modeled to elevate the still’s overall efficacy. The Fan Module is strategically situated to induce a targeted airflow pattern within the still, effectively disrupting the saturated boundary layer over the water surface and thus enhancing evaporation rates. It is waterproof which operates at 3000 rpm, DC 12 V. The fan speed of 3000 RPM in the solar still was selected based on experimental optimization to achieve effective turbulence for forced evaporation. The air mass flow rate was determined as 0.051 kg/s, with a corresponding volumetric flow rate of 0.040 m³/s (84.75 cfm).The speed controller has a specification of DC 6–60 V PWM Motor. Concurrently, the Ultrasonic Humidifier Module functions autonomously, generating a fine mist via ultrasonic vibrations that increases vapor concentration within the enclosure, consequently expediting condensation upon the inner glass surface. It has the capacity to release a moisture of 350 ml/h which has a specification AC 110–240 V. The 350 ml/h capacity represents an experimentally optimized balance, delivering enhanced evaporation and distillate yield while maintaining stable thermal conditions within the still, thus maximizing overall system performance. Each component was precisely dimensioned and integrally incorporated into the structural framework in a modular manner, permitting independent testing of individual parts and seamless integration into the primary solar still architecture without necessitating direct alterations, irrespective of the employed enhancement. Figure 2A shows the schematic sketch of the model, Figure 2B gives the dimensions of the model with the thermocouple position and Table 1 gives the specifications of the equipment’s used to build the solar still and its cost analysis.

**Table 1:**
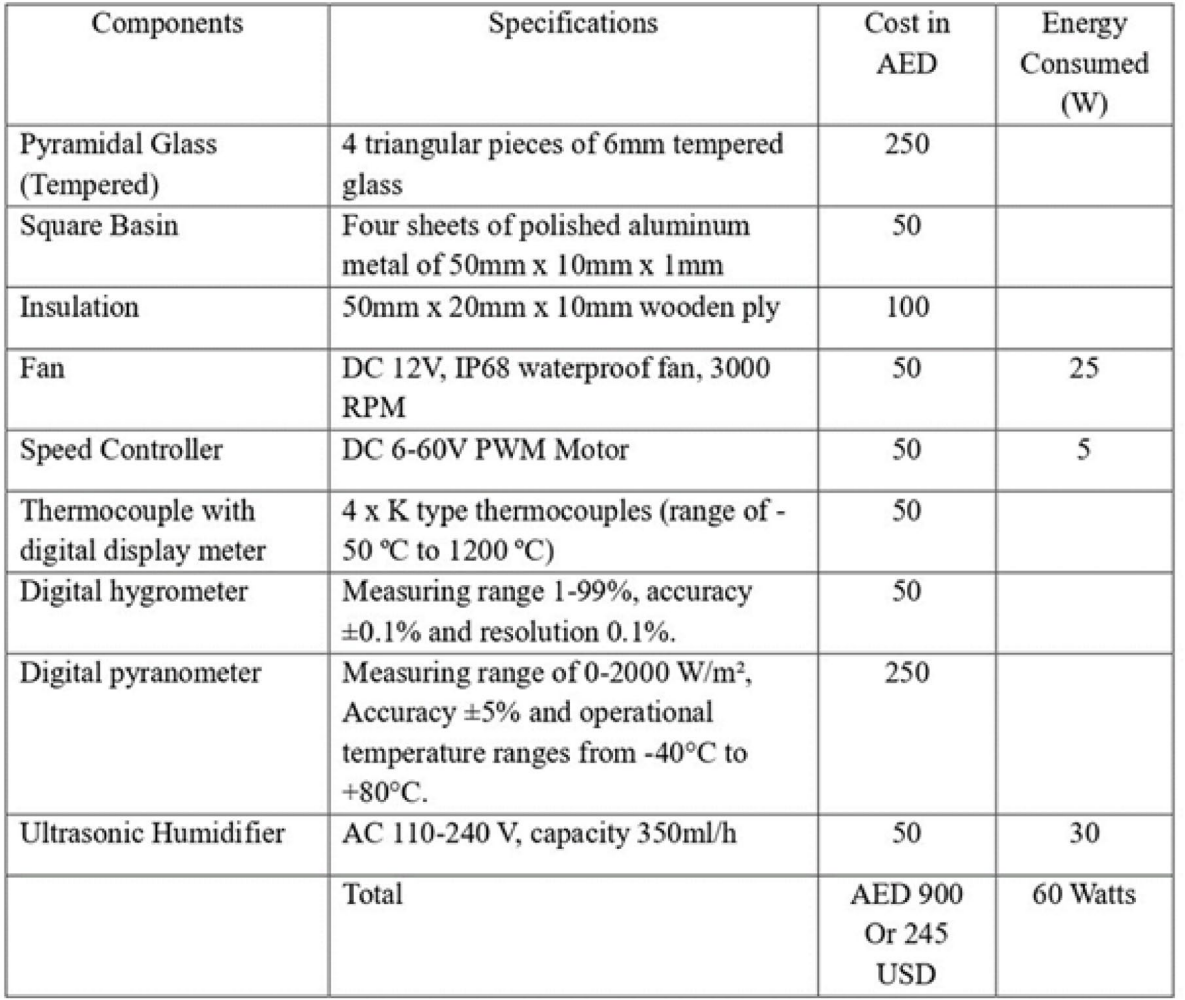
Components and their specifications along, cost and energy consumption.

### Working of pyramidal solar still

**Fig. 3:**
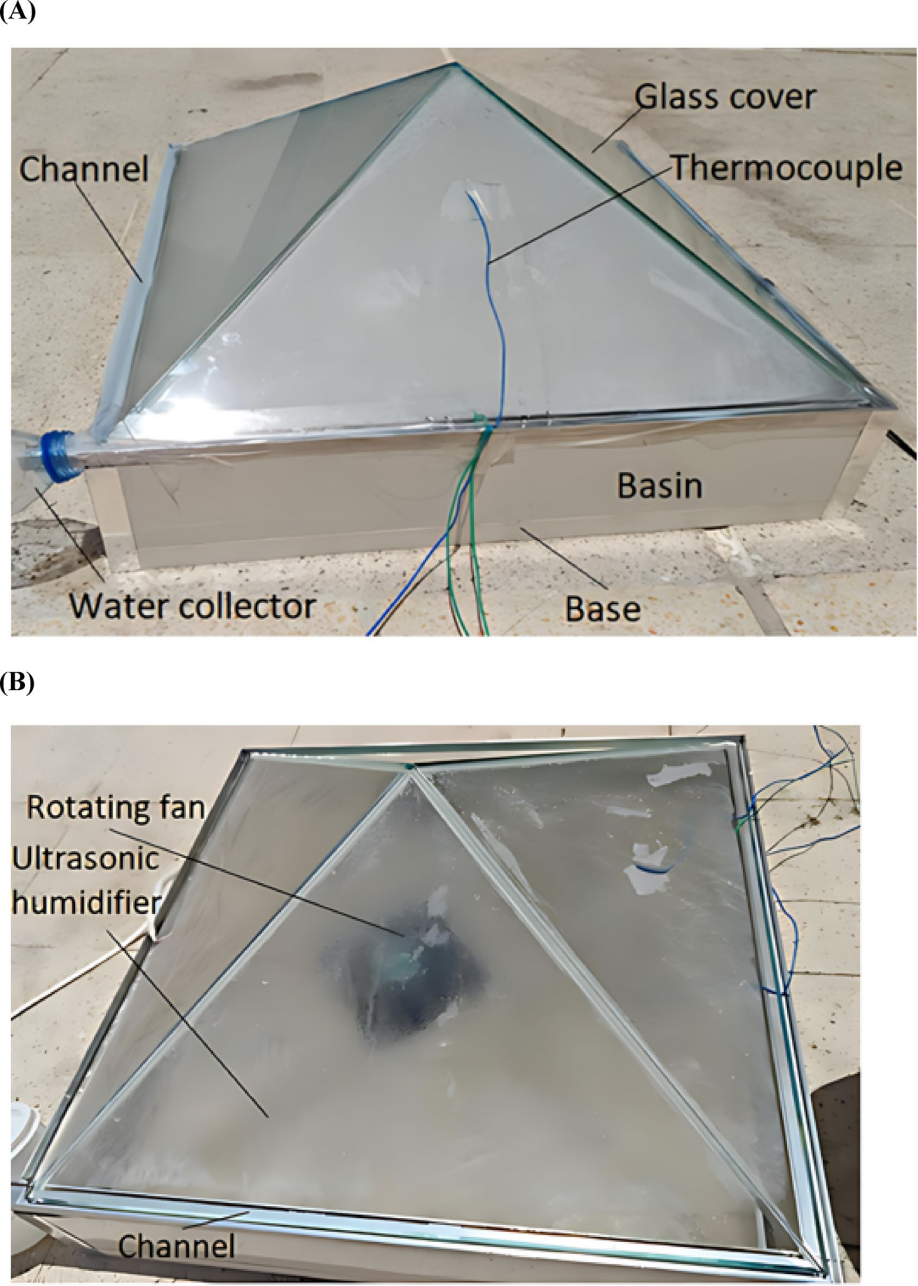
(**A**). Experimental test risk. (**B**). Solar still shows the rotating fan and ultrasonic humidifier.

A solar still equipped with rotating fan and an ultrasonic humidifier enhances the natural evaporation and condensation process through active mechanisms. Outdoor experiments were performed in real environmental conditions of Dubai, United Arab Emirates, during the month of May. Testing was performed daily from 11:00 AM-4:00 PM, a period selected to coincide with peak solar irradiance, ensuring consistent and optimal sunlight exposure for accurate performance evaluation. A digital pyranometer measures the solar irradiance during tests. Saline water was added to each still at a uniform depth of 2 cm, a shallow level chosen to maximize the surface-to-volume ratio and facilitate faster evaporation whereas deeper water reduces the evaporation. Solar radiation enters through transparent pyramidal top cover, heating water in the basin. Tests were conducted independently under three different conditions: static, dynamic (with a rotating fan), and ultrasonic humidifier-assisted modes. Active components, if used, should be timed carefully: a fan can be operated during peak sunlight hours (11 AM–4 PM) to boost vapor circulation, while an ultrasonic humidifier is best run in controlled cycles during high insolation to maximize output without excessive energy use. In the static configuration, water evaporated naturally through passive exposure to ambient solar heat. In the dynamic setup, the rotating fan enhanced evaporation by circulating warm air and disrupting the saturated boundary layer above the water surface. By dipping the fan in water, localized turbulence is created at the air–water interface, continuously disturbing the surface layer and breaking the thermal and saturated vapor boundary layers that normally restrict evaporation. This direct contact enhances the interaction between warm water and moving air, leading to a higher evaporation rate compared to positioning the fan near the cover, where its effect would be limited to air circulation alone In the third case, the ultrasonic humidifier generated a fine mist by vibrating water at high frequencies, increasing the vapor concentration within the chamber. This elevated humidity, combined with improved air circulation and thermal energy, accelerated and sustained the evaporation process. The resulting vapor condensed on cooler inside surface of glass cover & flowed into collection channels as purified water. The use of the fan and humidifier notably enhanced heat and mass transfer, thereby increasing overall efficiency & distilled water output of solar still. The performance of all three configurations was comparatively analyzed, graphically represented, and discussed in detail. Figure 3A and B shows the experimental test rig and the solar still showing the fan and ultrasonic humidifier.

### Instrumentation

**Fig. 4:**
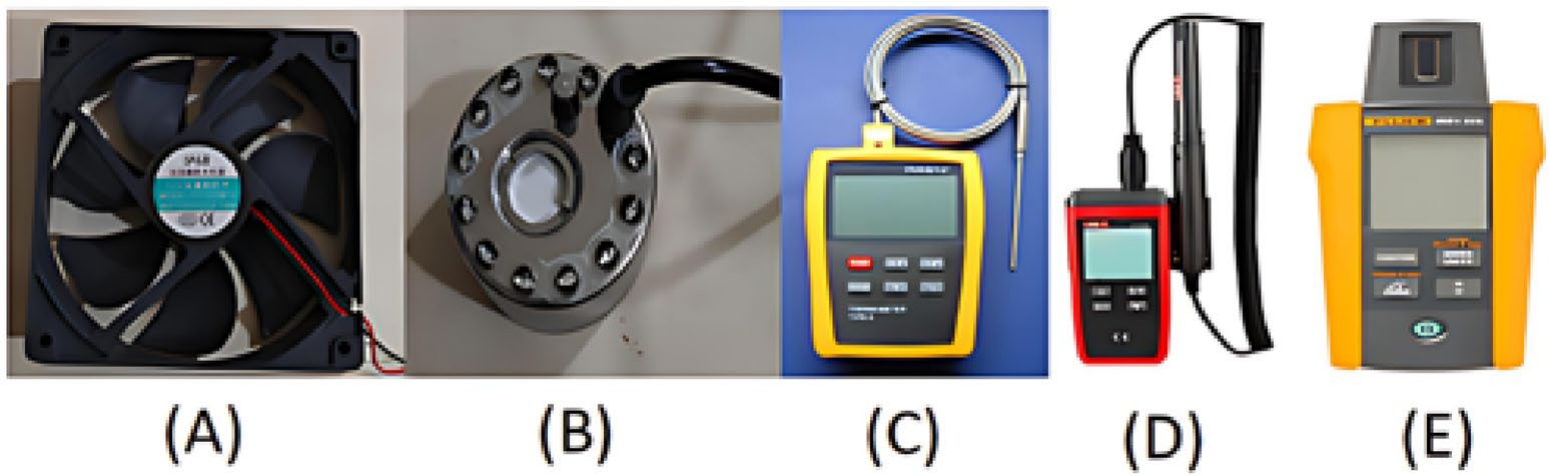
(**A**) Rotating fan (**B**) Ultrasonic humidifier (**C**) K type thermocouple (**D**) Hygrometer (**E**) Pyranometer.

A digital thermometer system utilizing K-type thermocouples was used to monitor thermal performance of solar stills, chosen for their reliability, durability, and precision. Four thermocouples were strategically placed at critical locations: the inside surface of the glass cover, basin liner, water in the basin, & surrounding ambient air. Temperature readings were recorded at hourly intervals, providing valuable insights into thermal gradients and the dynamic behavior of the system, thus enabling a comprehensive performance evaluation. The K-type thermocouples featured a temperature range of −50–1200 °C, with accuracy of ± 0.1 °C & resolution of 0.1 °C. Relative humidity (RH) was measured using a hygrometer, offering a measuring range of 1% to 99%, with a resolution of 0.1% and the same accuracy level. Solar irradiance was monitored using a pyranometer, capable of measuring within a range of 0–2000 W/m², featuring high accuracy of ± 5% and an operational temperature range between − 40 °C to + 80 °C. Figure 4 shows photographs of fan, ultrasonic humidifier and instruments used in the experimental set up.

### Uncertainty analysis and experimental conditions

The Root Sum of Squares (RSS) uncertainty technique is a widely used that evaluates overall results by considering independent variables. Commonly used in thermal and solar energy studies, such as solar stills, it accurately assesses performance metrics influenced by factors like temperature, irradiance, and water mass. RSS also supports sensitivity analysis, improving experimental design and data reliability. Its versatility makes it a standard tool across engineering, environmental monitoring, and instrumentation in both research and industry.

Let Y be the uncertainty intervals, G be the function, X be the independent variable and Y_G_ be the overall uncertainty. The total uncertainty value is evaluated using the general RSS equation as shown in (7).

7 $$\:{{\text{Y}}_{G}\:=\:\left[{\left(\frac{\delta\:G}{\delta\:{X}_{1}}\:{Y}_{1}\right)}^{2}+\:{\left(\frac{\delta\:G}{\delta\:{X}_{2}}\:{Y}_{2}\right)}^{2}+{\left(\frac{\delta\:G}{\delta\:{X}_{3}}\:{Y}_{3}\right)}^{2}+\dots\:\dots\:\dots\:\dots\:.\:+{\left(\frac{\delta\:G}{\delta\:{X}_{4}}\:{Y}_{n}\right)}^{2}\right]}^{0.5}$$

Following the analysis, total uncertainty associated with evaluating the output of the solar still was evaluated and is presented in Table 2 and the Table 3 gives the details of the experimental conditions.

**Table 2:**
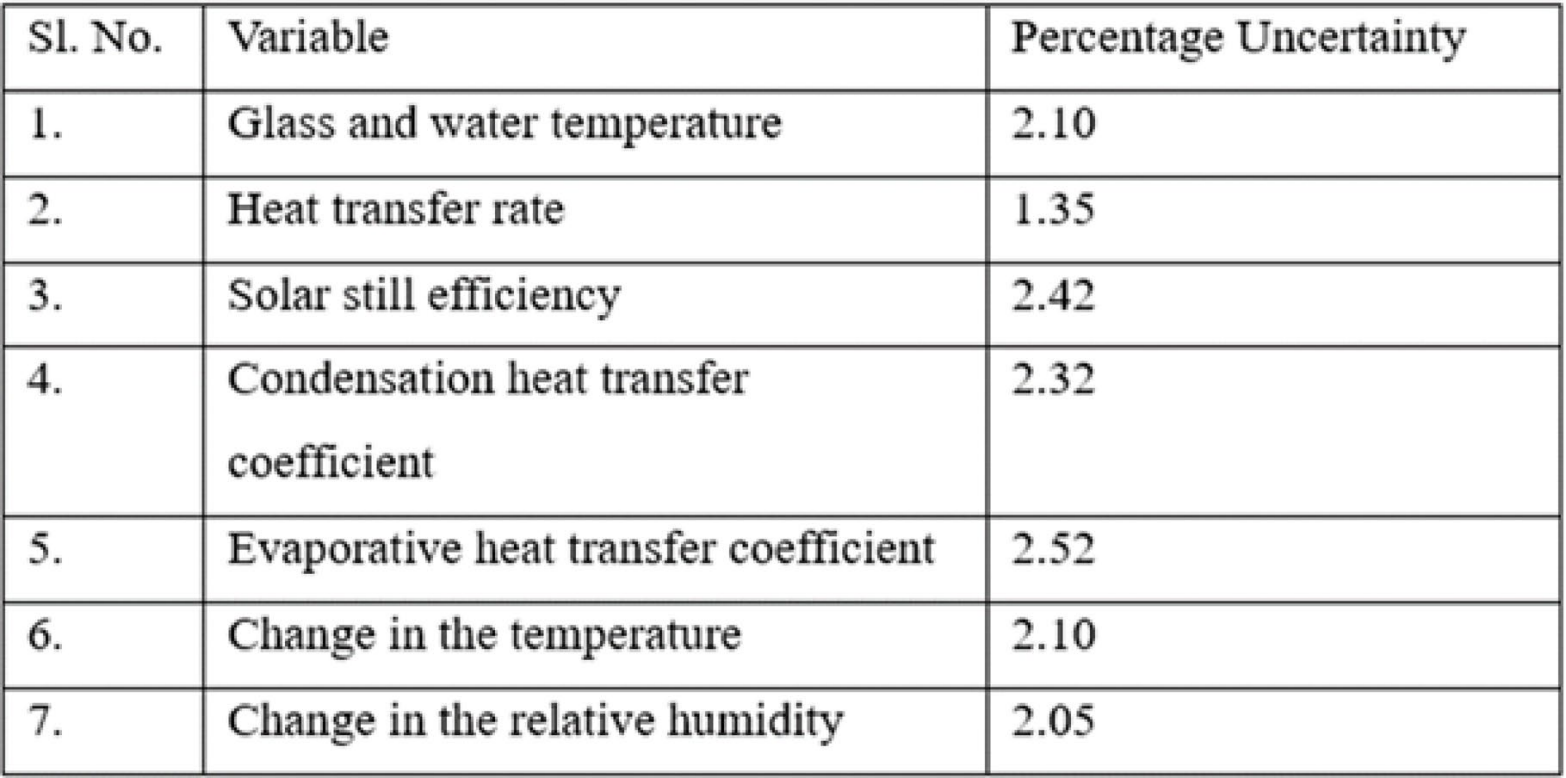
Error present while evaluating output of pyramidical solar still.

**Table 3:**
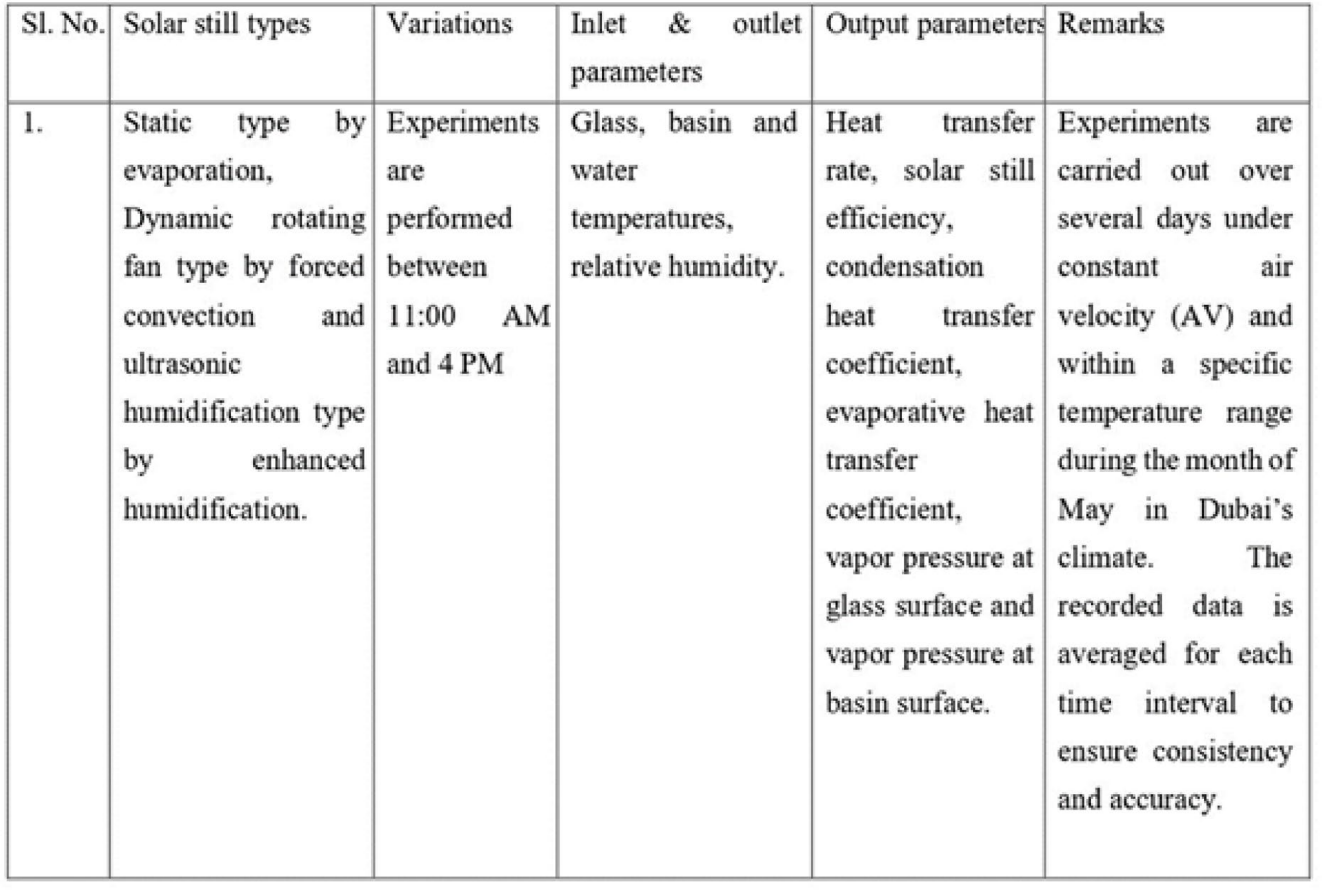
Empirical conditions to evaluate the output of pyramidical solar still.

## Experimental conditions and procedure

### Experimental procedure

The experiments were conducted outdoors in Dubai, UAE, during May under real climatic conditions. Tests were carried out daily between 11:00 AM and 4:00 PM, corresponding to the period of maximum solar irradiance, which was measured using a digital pyranometer. Each still was filled with saline water at a uniform depth of 2 cm to optimize the surface-to-volume ratio and accelerate evaporation. Three operating modes were examined: static, dynamic (fan-assisted), and ultrasonic humidifier-assisted. In all cases, the vapor condensed on the inner glass surface and was collected as distilled water, which was measured for yield. The basin water, surface, and glass temperatures, as well as the internal humidity, were regularly recorded. Performance parameters were evaluated using Eqs. (1)– (6). Overall, the incorporation of the fan and ultrasonic humidifier significantly enhanced heat and mass transfer, leading to higher efficiency and greater distillate yield compared to the static configuration.

## Results and discussion

Tests were performed between 11:00 AM & 4:00 PM during the summer month of May under the climatic conditions of Dubai to coincide with the period of peak solar irradiance, which is pivotal for maximizing the thermal efficiency of the system. During this interval, the sun approaches its zenith, delivering the highest intensity of solar radiation, which elevates the temperatures of the basin, water, and glass, thereby augmenting evaporation and condensation rates. This temporal window also ensures a stable and representative thermal profile, effectively capturing the transient heat transfer dynamics of the still under optimal operating conditions. Performing experiments within these hours enables a robust evaluation of the still’s performance, encompassing both passive and active enhancement strategies, while adhering to established protocols in solar distillation research to facilitate meaningful comparison and reproducibility. Three types of solar stills were examined: the static type, the dynamic type with a rotating fan, and the ultrasonic humidifier-assisted type. Performance parameters were calculated using the experimental data and Eq. (1) to (6), and the results were graphically represented. This section highlights the variation of key performance parameters throughout the day, offering a comparative analysis of each configuration’s efficiency.

### Basin, glass and water temperature

**Fig. 5:**
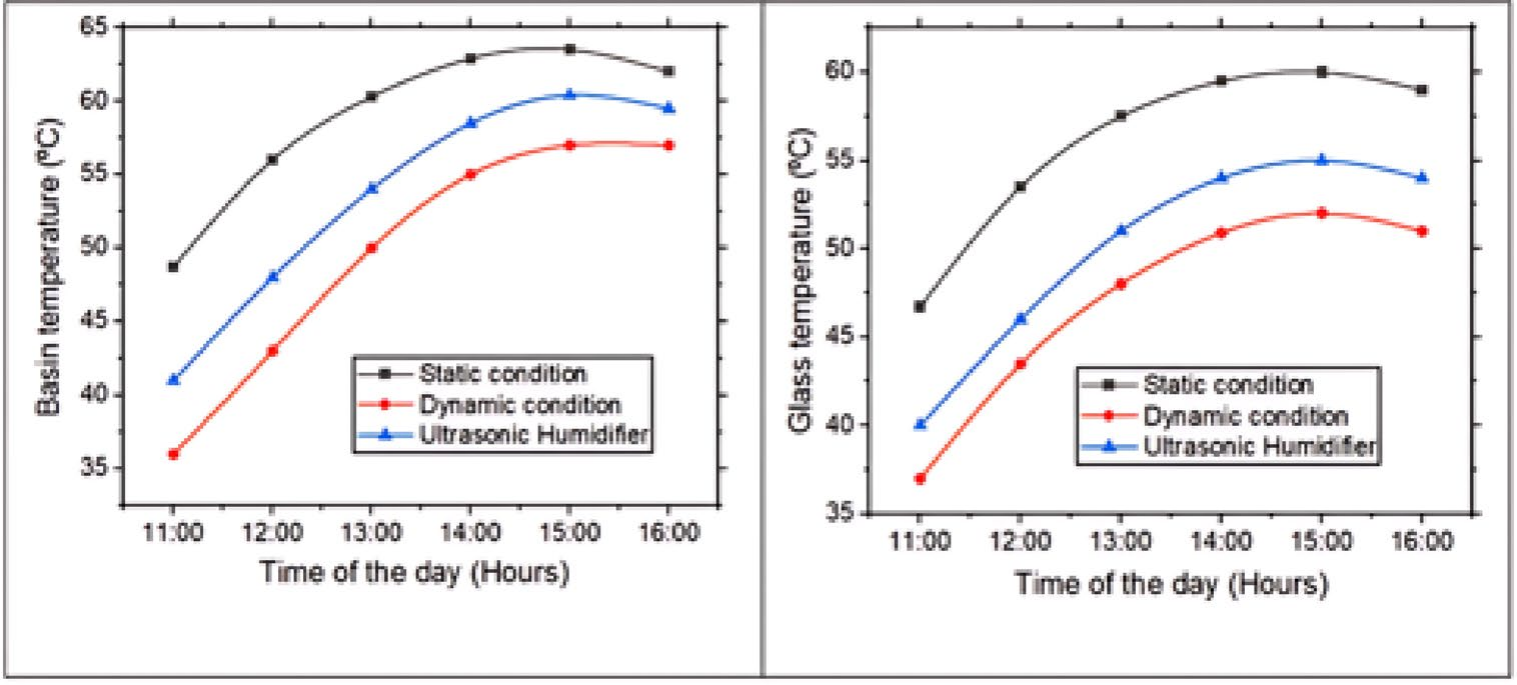
Basin and glass temperature with time of day.

**Fig. 6:**
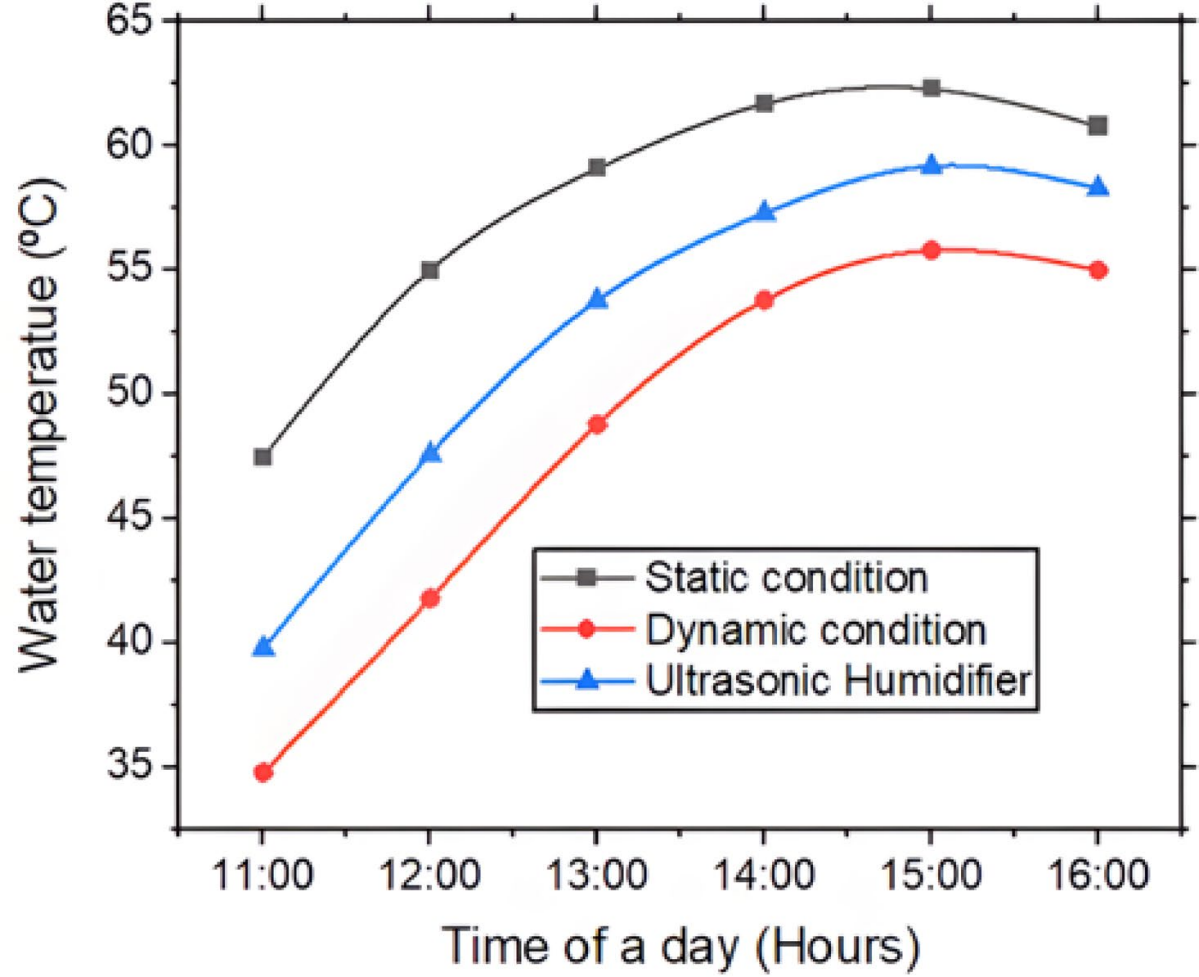
Water temperature with time of day.

Figures 5 and 6 gives variation of basin, glass & water temperature with the time of the day. The rise and slight drop in basin, glass, and water temperatures in a pyramidal solar still from 11:00 AM to 3:00 PM can be explained by basic principles of solar radiation and heat transfer. From 11:00 AM to around 3:00 PM, solar irradiance increases and typically peaks near solar noon (approximately 12:00 to 1:00 PM), when the sun is at highest point in the sky. During this period, pyramidal glass cover allows maximum solar radiation to enter the still, which is absorbed by blackened basin liner and transferred to water through conduction & convection. As a result, temperatures of basin, water, and glass all rise due to the continuous accumulation of thermal energy. However, after 3:00 PM, solar intensity begins to drop as sun moves lower in the sky, reducing the amount of energy entering the system. The reduced irradiance means less heat is absorbed, and at the same time, the system begins to lose heat to the environment through convection (with cooler ambient air) and radiation. As the rate of heat loss starts to exceed rate of heat gain, temperatures of the basin, glass, and water exhibit a slight decline. Thus, the temperature profile observed in a pyramidal solar still reflects the balance between incoming solar energy and heat losses, governed by the principles of radiation, conduction, and convection throughout the day. In a static solar still the basin, glass and water temperature increased by 28.33%, 28.47% and 31.15% for the time 11 AM to 3 PM.

The observed temperature rise being highest in the static solar still, moderate in the ultrasonic humidifier-assisted still, and lowest in the dynamic rotary fan-based still can be related to differences in heat and mass transfer mechanisms within each system. In the static solar still, there is minimal internal air movement, allowing the absorbed solar energy to primarily heat the basin, water & glass, resulting in a greater temperature build-up due to lower heat dissipation. In contrast, the ultrasonic humidifier introduces fine water mist into the chamber, which absorbs some of the thermal energy for phase change, thereby moderating the temperature rise while enhancing evaporation. In the dynamic rotary fan system, the continuous circulation of air disrupts the thermal boundary layers and promotes forced convection, which enhances heat transfer away from the water and internal surfaces. This increased convective heat loss prevents the temperatures from rising as much as in the static case. Therefore, the more active the heat and mass transfer mechanisms, the more the system favors evaporation over temperature accumulation, explaining the observed temperature trends. At a peak temperature of 3 PM, static solar still gave 11.40% higher basin temperature, 15.38% higher glass temperature and 11.64% higher water temperature compared to dynamic solar still. Solar still with ultrasonic humidifier yielded intermediate results.

### Partial vapor pressure of basin & glass

**Fig. 7:**
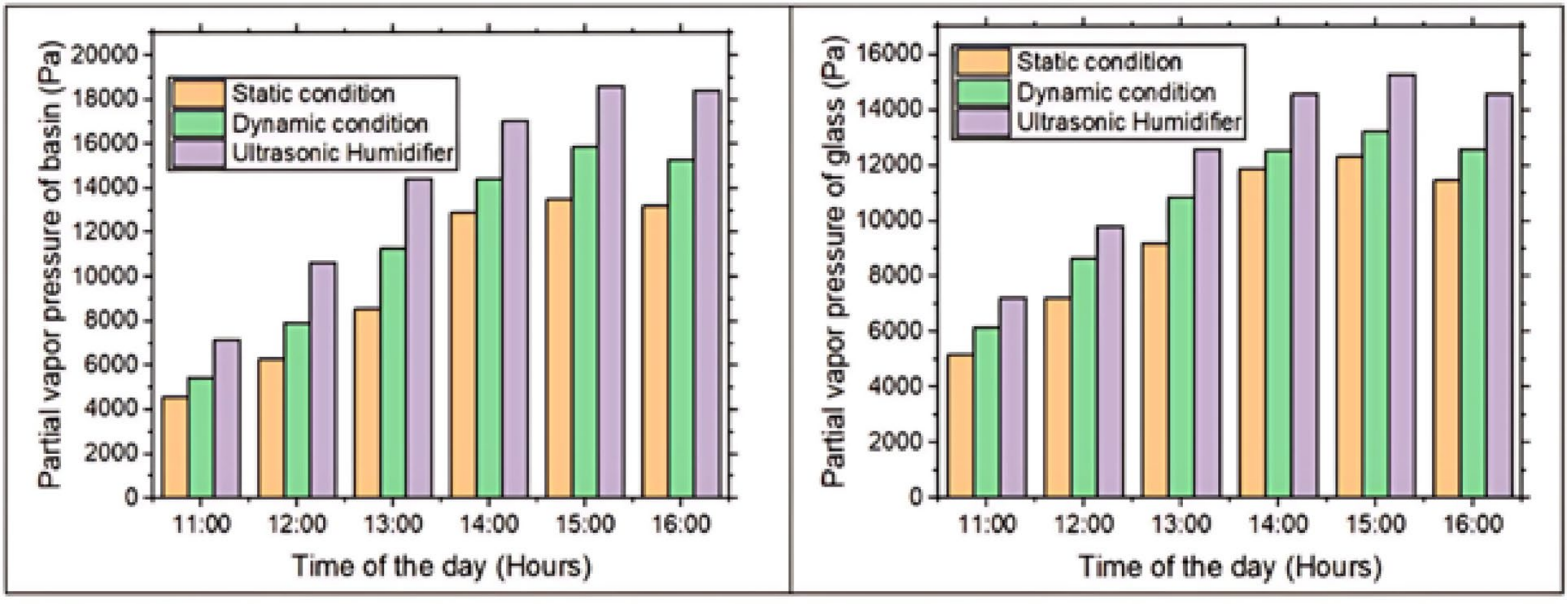
Partial vapor pressure of basin and glass with the time of day.

Figure 7 shows the variation in partial vapor pressure of basin & glass surfaces of a solar still between 11:00 AM and 4:00 PM. From 11:00 AM-3:00 PM, the increasing solar radiation intensity elevates temperature of basin water, thereby enhancing kinetic energy of its molecules. As molecular agitation intensifies, a larger number of water molecules overcome intermolecular forces and escape into the vapor phase, resulting in a rise in partial vapor pressure above basin. Concurrently, inner surface of glass, warmed by both incident solar radiation and contact with humid air, supports elevated vapor pressure, even as it serves as a site for condensation. This upward trend continues until approximately 3:00 PM, when solar input peaks. Beyond this point, diminishing solar irradiance leads to a gradual decline in temperature. As thermal energy decreases, molecular motion slows, reducing the evaporation rate & consequently, partial vapor pressure above both basin and the glass surfaces. This temporal behavior exemplifies the fundamental relationship between temperature, molecular kinetic energy, and vapor pressure in a closed thermal system. Maximum value of the partial vapor pressure of basin & glass is found to be 18626.71 Pa and 15276.53 Pa.

The observed trend in partial vapor pressure—highest in the ultrasonic humidifier-based solar still, followed by the dynamic fan-assisted system, and lowest in the static configuration—can be attributed to the fundamental principles of heat & mass transfer. In ultrasonic humidifier-based still, high-frequency oscillations atomize the water into a fine mist, substantially increasing the number of water molecules transitioning into vapor phase. This results in a significantly elevated vapor density within the chamber, thereby raising the partial vapor pressure near both basin and inside glass surface. The dynamic system, equipped with a rotating fan, enhances evaporation by disrupting the saturated boundary layer above the water surface and promoting forced convection, which facilitates the escape of vapor molecules and moderately increases vapor pressure. In contrast, static still operates solely on natural convection and passive heating, leading to a comparatively slower evaporation rate and the lowest partial vapor pressure. This hierarchical variation directly reflects each system’s capacity to promote phase transition from liquid to vapor. At the peak heating period around 3:00 PM, the ultrasonic humidifier-based solar still recorded markedly higher partial vapor pressures—38.04% at the basin surface and 23.87% at the glass surface—compared to the static configuration, underscoring the effectiveness of active enhancement in accelerating vapor generation.

### Condensation and evaporative heat transfer coefficient

**Fig. 8:**
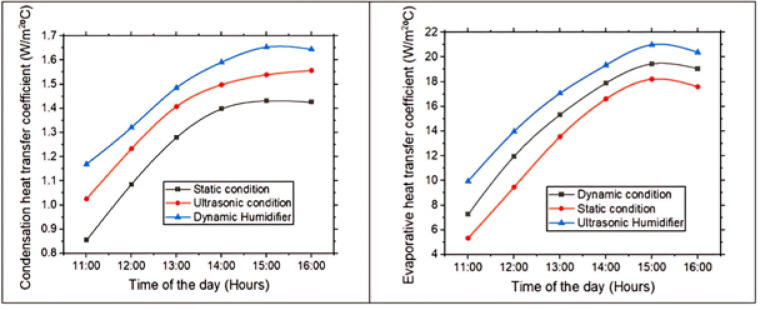
Condensation and evaporative heat transfer coefficient with time of day.

Figure 8 illustrates variation of condensation & evaporative heat transfer coefficients over time. The evaporative heat transfer coefficient represents the rate of latent heat absorption during the evaporation process, while the condensation heat transfer coefficient denotes the rate of latent heat release during condensation. Both are influenced by temperature gradients, surface conditions, and fluid properties, making them crucial parameters for analyzing phase-change phenomena in solar stills. In a pyramidal solar still, these coefficients increase steadily from 11 AM to 3 PM as rising solar intensity and ambient temperature enhance thermal gradients between basin water & glass cover. This results in high water temperatures and increased vapor generation, thereby elevating evaporative heat transfer coefficient. Simultaneously, relatively cooler glass covers facilitate efficient condensation, raising the condensation heat transfer coefficient. After 3 PM, the declining solar radiation reduces water temperature and vapor production, while the glass cools further, collectively diminishing the temperature gradients. Consequently, both coefficients decrease, highlighting the strong dependence of phase-change heat transfer on solar energy availability and internal thermal differentials in the still. For the increase in the time from 11 AM to 3 PM, there is a rise in the condensation heat transfer & evaporative heat transfer coefficient by 42.24% and 111.16%. The dynamic solar still showed a higher condensation heat transfer coefficient than the ultrasonic humidifier and static stills due to increased fluid turbulence from the rotating fan, which enhances vapor removal and temperature difference, accelerating condensation. This improved convection reduces thermal resistance on the glass, promoting faster heat release. In contrast, the static still lacks airflow, and the ultrasonic humidifier boosts vapor generation but not vapor removal, making the dynamic system more efficient. The ultrasonic humidifier raised evaporative heat transfer coefficient by generating fine vapor droplets, increasing vapor concentration and mass transfer near the water surface, thereby enhancing evaporation more effectively than dynamic and static stills. In a solar still, evaporative heat transfer coefficient is typically higher than condensation coefficient because evaporation occurs at the warm basin with ample solar heat, while condensation on the cooler glass is limited by smaller temperature differences and condensate resistance, making condensation the rate-limiting step. The maximum values of the condensation and evaporative heat transfer coefficients were determined as 1.65 W/m²·°C and 20.99 W/m²·°C, respectively.

In a pyramidal solar still, the evaporative heat transfer coefficient is greater than the condensation coefficient due to the physics of phase change and thermal gradients. Evaporation is enhanced by direct solar heating of the basin water, which creates a significant temperature and vapor pressure difference between the hot water surface and the air just above it. This strong gradient promotes rapid molecular escape from the liquid, resulting in a higher energy transfer rate per unit area. In contrast, condensation on the cooler glass cover occurs with a smaller temperature difference between vapor and surface, while the buildup of droplets or thin films adds resistance to heat flow, reducing the condensation coefficient. Hence, the larger driving force and lower resistance at the evaporation surface explain why the evaporative coefficient exceeds the condensation coefficient in a pyramidal solar still. Similar trends are also seen in the literature^9^.

### Heat transfer rate and solar still efficiency

**Fig. 9:**
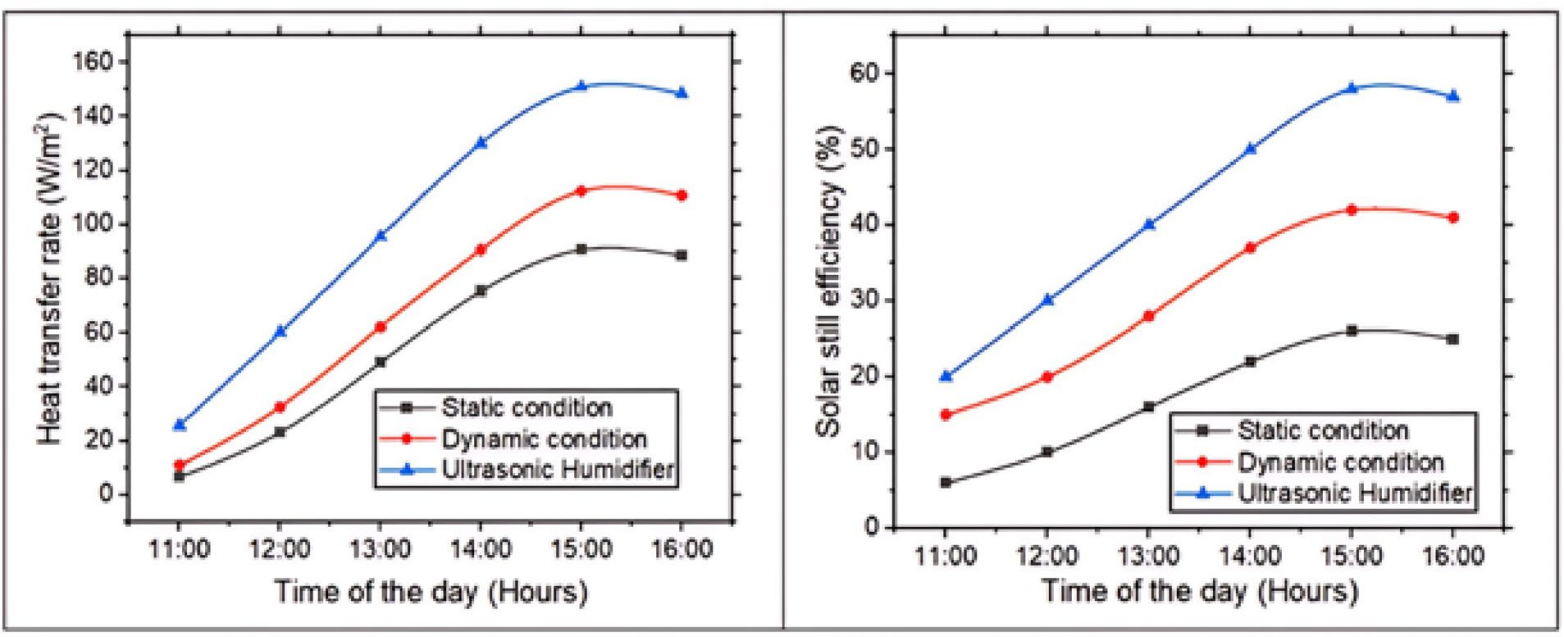
Heat transfer rate and solar still efficiency with time of day.

Figure 9 presents the change in heat transfer rate and efficiency of the pyramidal solar still between 11:00 AM & 4:00 PM. As solar radiation intensifies, basin water temperature rises, creating a thermal gradient between warmer water surface & cooler glass cover. This temperature difference induces buoyancy-driven natural convection currents within the enclosed air–vapor mixture, facilitating the upward movement of warm, moisture-laden air. From 11:00 AM to 3:00 PM, the strength of these convective flows increases with the rising thermal gradient, enhancing vapor transport toward the condensing surface and thereby improving both heat and mass transfer. This efficient internal circulation supports sustained evaporation and condensation, resulting in elevated system performance. After 3:00 PM, the declining solar irradiance weakens the temperature gradient, diminishing convective activity and reducing overall thermal performance. Accordingly, the solar still’s efficiency is strongly governed by the dynamics of thermally induced convection. The peak heat transfer rate and efficiency observed were 150.96 W/m² and 58%, respectively.

In comparison, the solar still integrated with an ultrasonic humidifier demonstrated superior thermal behavior. By generating fine water droplets through high-frequency oscillations, the humidifier markedly increases the evaporative surface area, leading to a rapid rise in vapor concentration within the chamber. This intensified vapor presence enhances the vapor pressure gradient between the basin and the condensing surface, while also driving more robust convective motion as warmer, saturated air rises and cooler air descends. In comparison, the fan-assisted system improved efficiency by inducing forced convection, which disrupted the saturated boundary layer above the water surface and promoted better air circulation, but its effect on vapor concentration was less pronounced than that of the humidifier. Unlike the fan-based configuration, which only improves air movement, or the static design, which relies solely on natural convection, the ultrasonic humidifier simultaneously amplifies both evaporation and vapor transport. This synergistic enhancement of internal fluid dynamics significantly boosts heat and mass transfer, thereby elevating efficiency. Static still, relying solely on passive solar heating, lacked both forced convection and artificial humidification, resulting in the slowest evaporation rate and lowest overall performance. Thus, the ultrasonic humidifier created the most favorable thermal and mass transfer conditions, yielding the highest distillate output and efficiency among the three configurations. Compared to static solar still, ultrasonic humidifier-based system achieved a 66.64% increase in heat transfer rate and a remarkable 123.07% improvement in overall efficiency.

### Distillate output

**Fig. 10:**
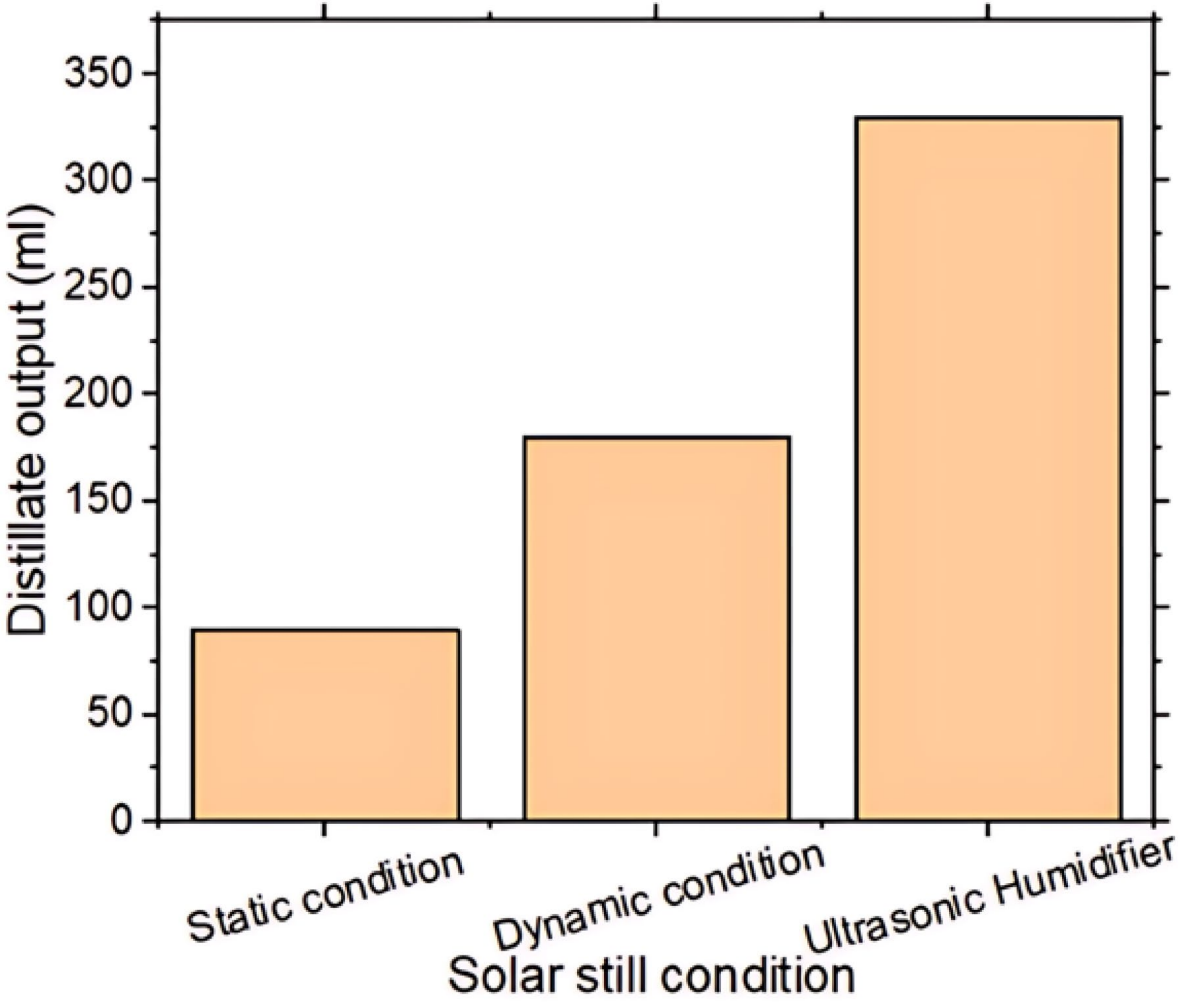
Distillate output vs. solar still condition.

Figure 10 illustrates the distillate output of the three solar still configurations. The ultrasonic humidifier-based solar still demonstrates highest distillate yield, surpassing both dynamic and static systems due to its advanced enhancement of evaporation and vapor saturation, governed by the principles of phase change and thermal energy transfer. The humidifier generates high-frequency oscillations that produce a fine mist of water droplets, substantially increasing the effective surface area for evaporation. This intensifies the rate at which water molecules transition to the vapor phase, thereby elevating vapor density within chamber. The heightened vapor pressure strengthens gradient between heated basin & cooler inside glass surface, resulting in more efficient and sustained condensation. In contrast, the dynamic solar still, though it enhances air movement through forced convection, does not promote vapor generation as effectively. The static still, relying solely on natural convection and passive solar heating, exhibits the lowest evaporation rate and vapor concentration. Consequently, the ultrasonic humidifier’s dual role in boosting both evaporation and vapor availability lead to significantly greater distillate output. Over the five-hour experimental period from 11:00 AM-4:00 PM, the ultrasonic humidifier-based solar still yielded 330 ml of distilled water, as compared to 180 ml from the dynamic still and 90 ml from the static still.

A payback analysis of the pyramidal solar still costing 900 AED, equipped with a 25 W fan and 30 W ultrasonic humidifier, was performed based on its measured yield. The system produces about 0.33 L/day (≈ 120 L/year or 0.12045 m³/year) while consuming 100.4 kWh/year of electricity. At 0.23 AED/kWh, the electricity cost is 23 AED/year, and with an additional 50 AED for maintenance, the total annual operating cost is about 73 AED. This output corresponds to 1 L every three days, or 120 L annually. Considering bottled water in the UAE costs about 3 AED per liter, the still generate savings of roughly 360 AED/year. Based on these values, the system requires around 2.5 years to recover the initial 900 AED investment, after which it begins to provide net savings. For comparison, a small desalination unit with a capital cost of 1500 AED produces 0.5 L/day (≈ 180 L/year) with annual operating costs of about 100 AED. At the same water valuation of 3 AED per liter, this unit yields savings of 540 AED/year, resulting in a payback period of about 3 years before generating net benefits.

Based on the experimental outcomes, the performance parameters for the static, dynamic rotary, and ultrasonic humidifier-based solar stills are compiled and presented in Table 4. Among the three configurations, the ultrasonic humidifier-based solar still exhibited superior results, outperforming both the static and dynamic types.

**Table 4:**
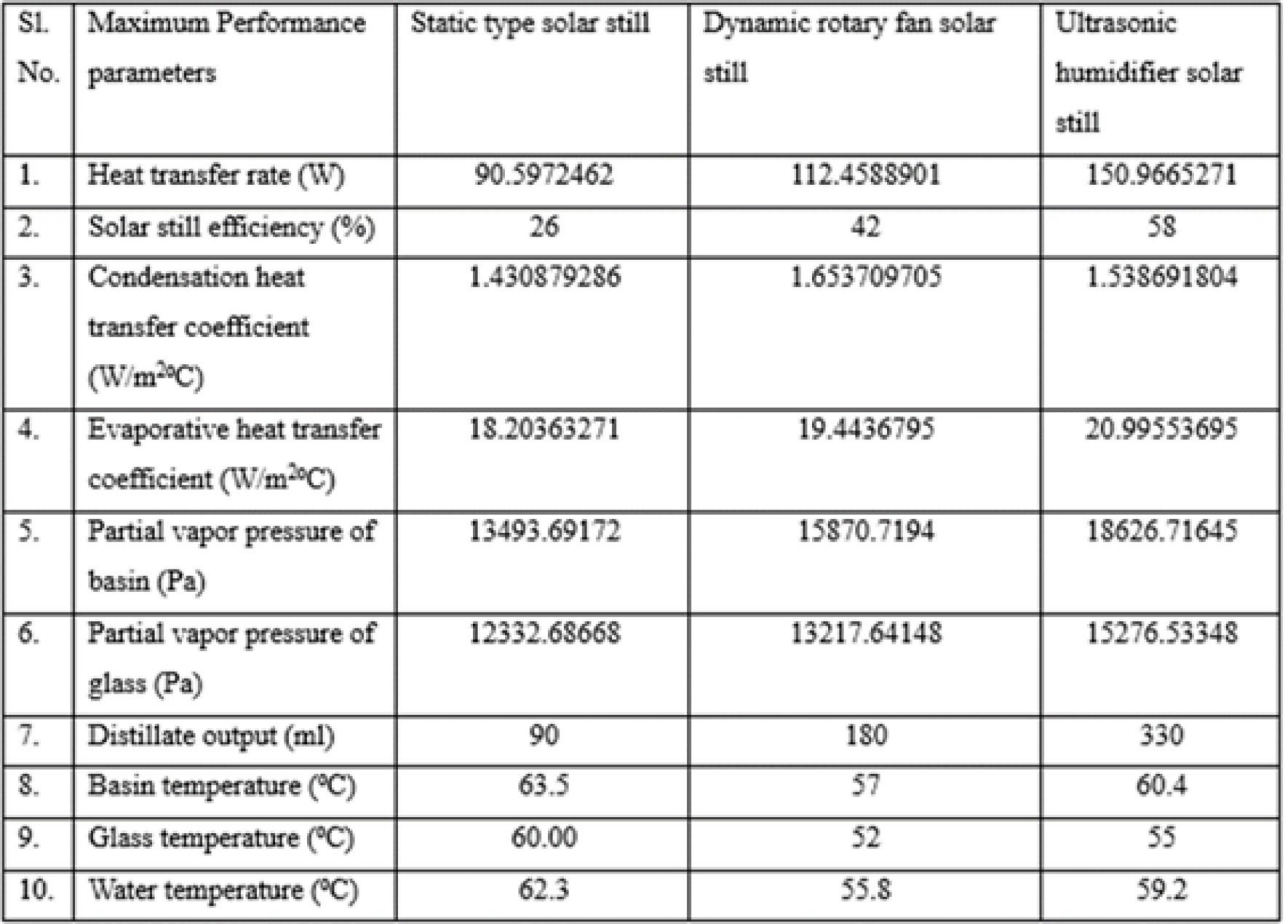
Experimental results of different configuration solar still.

### Scaling up a pyramidal solar still for community

level water production faces challenges such as high land and material requirements, complex thermal management, and increased maintenance due to fouling and cleaning needs. Structural stability and sealing become harder to ensure in larger units, while higher investment costs and reduced efficiency per unit area limit economic feasibility. Addressing these issues may require innovative designs, hybrid energy integration, or modular arrays of smaller stills instead of a single large structure.

### Importance of regular maintenance

In practical use, a pyramidal solar still faces performance losses due to condensation on the inner glass surface. While essential for water collection, excessive droplet or film formation scatters sunlight and reduces heat input for evaporation. Coalesced droplets can streak the glass, further lowering optical efficiency, while dust, salt scaling, and fouling worsen the problem over time, demanding regular cleaning. Mitigation strategies such as surface coatings, optimized glass tilt, or controlled heating/ventilation help maintain transparency and ensure consistent freshwater productivity.

The data for solar irradiance at different times, along with glass temperature, water temperature, and solar still efficiency, are presented in Table 5 below.

**Table 5:**
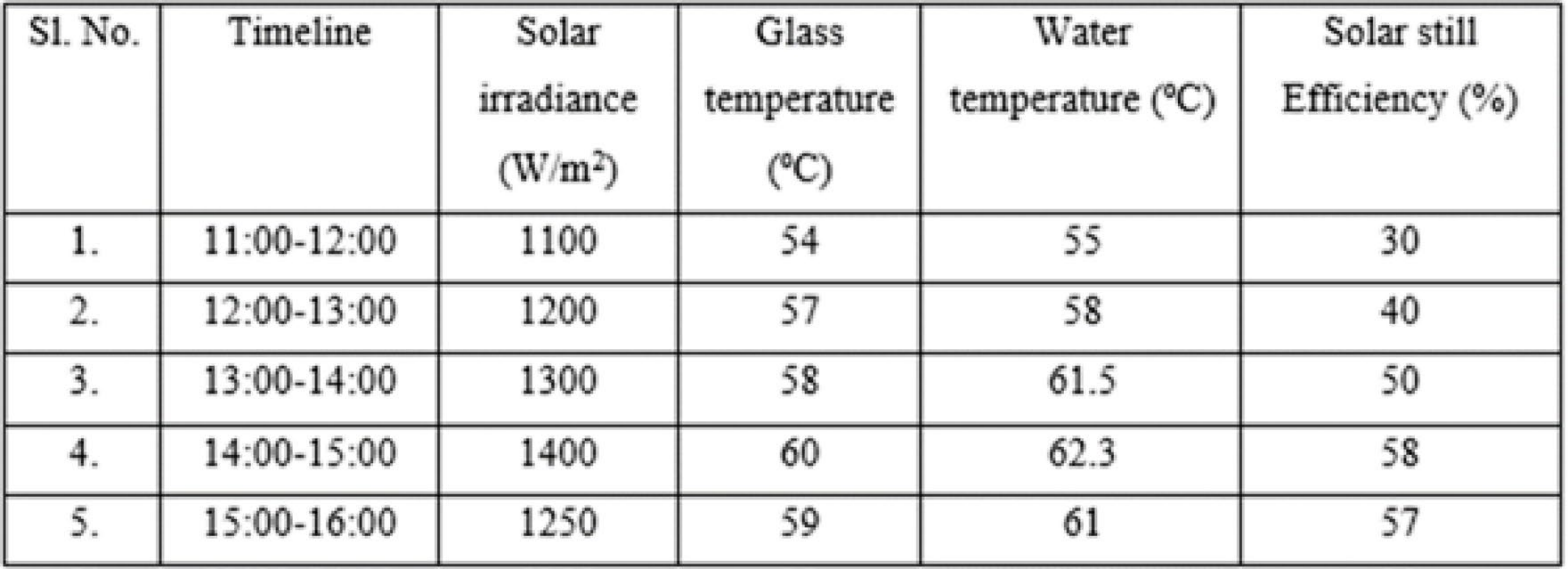
Relationship between the solar irradiance, temperature and efficiency.

## Validation of the results

Table 6^26–33^ gives a comparative analysis between the experimental results & values reported in the literature. Validating key parameters such as solar still efficiency and distillate output against established studies ensures the accuracy and credibility of the findings. This comparison sets a benchmark for determining performance improvements, highlighting the impact of design modifications and environmental conditions. It also reinforces the methodological robustness of the study, supports the reproducibility of results, and enhances the overall contribution to the field of renewable energy research. Results indicated that results obtained by the current research paper are at par with that of the literature values. Hence the results are validated.

**Table 6:**
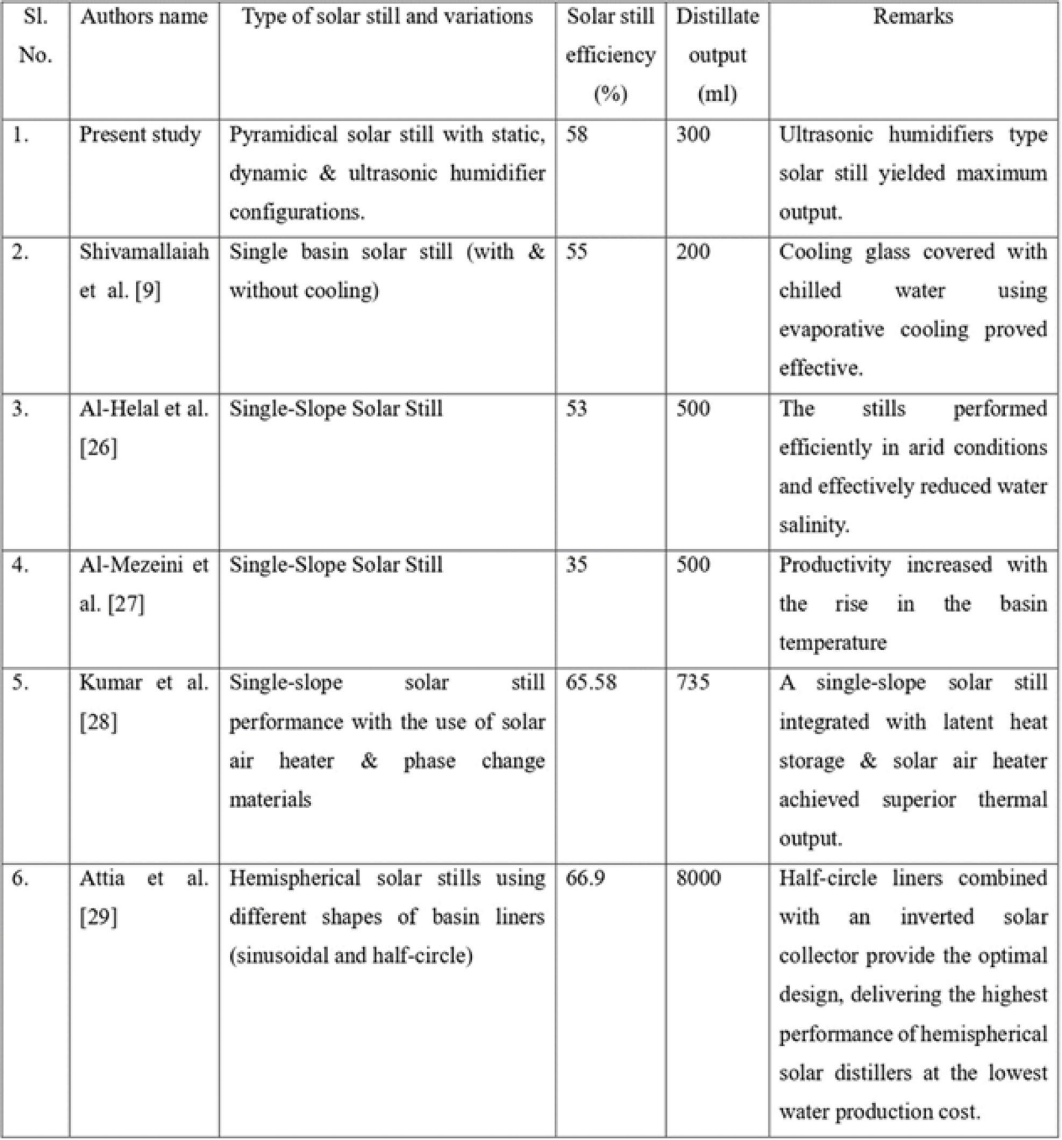

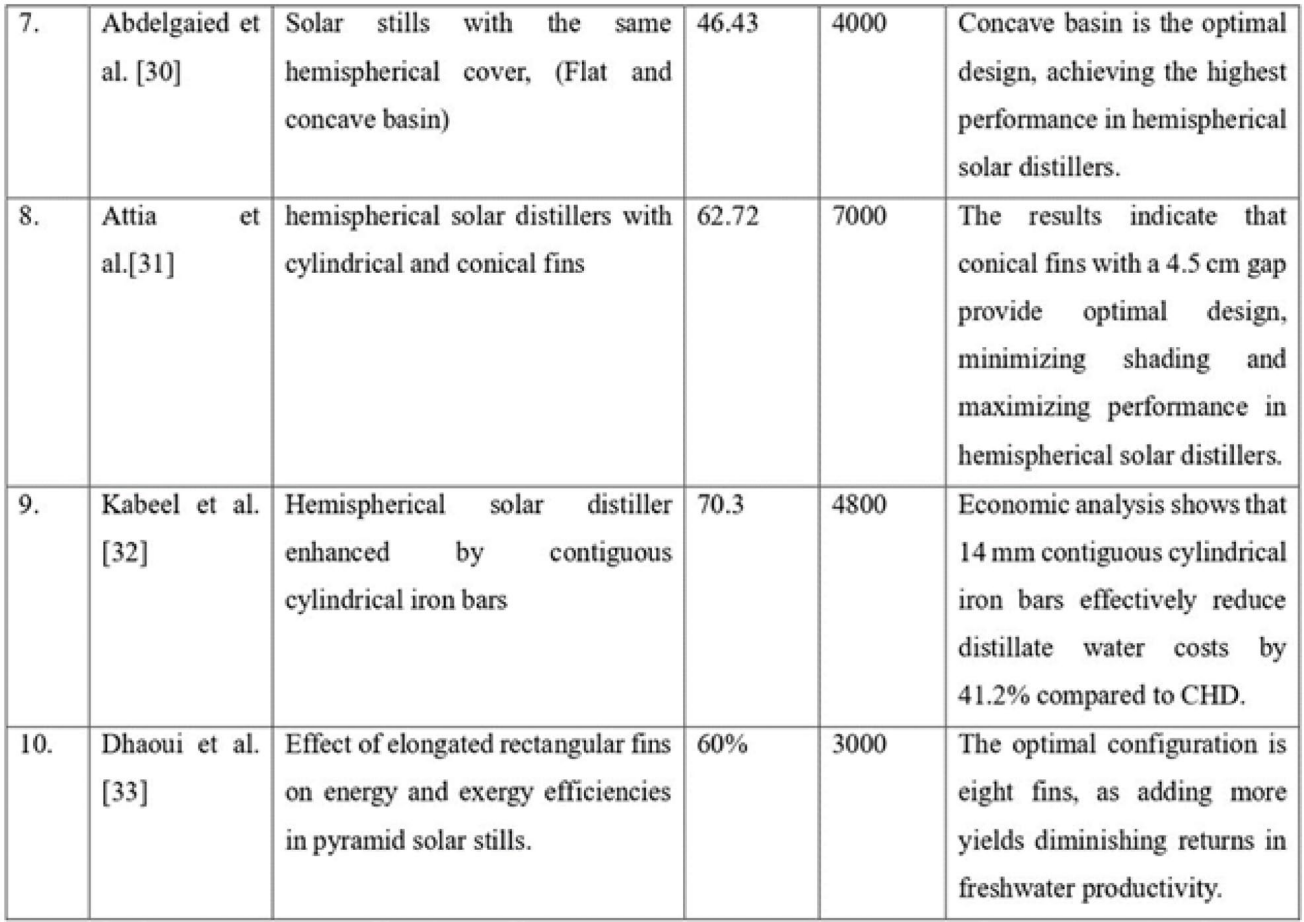
Validation of the current results with literature values.

### Water quality check and carbon footprint

Water quality tests were conducted to identify the presence of various chemicals in the sample. Table 7 presents the results, confirming that the condensate from the pyramidal solar still meets established water quality standards.

**Table 7:**
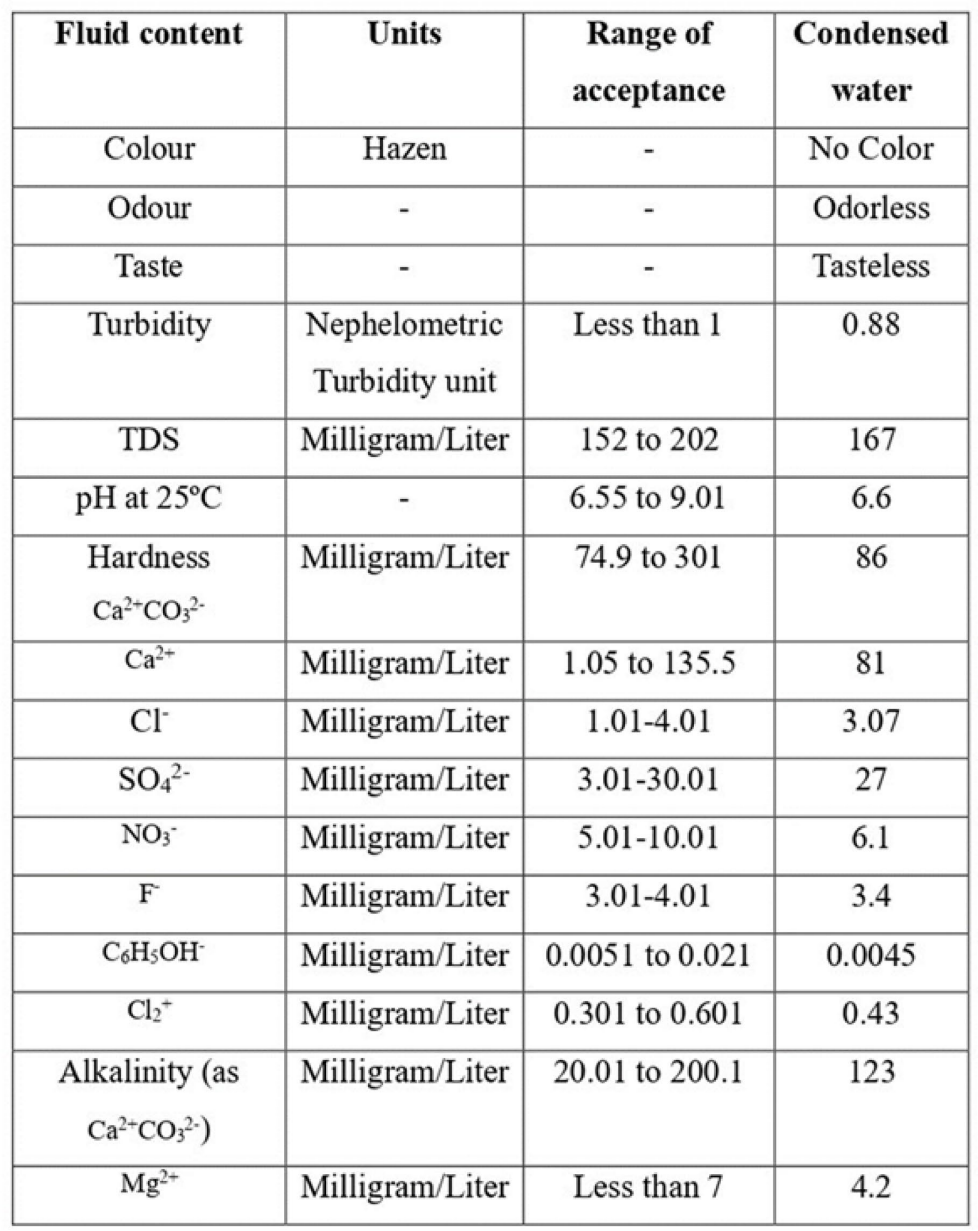
Water quality check.

The CO₂ emissions of a pyramidal solar still are estimated via a life-cycle assessment, accounting for both embodied and operational impacts. Embodied carbon from materials like glass, metal, and insulation is summed and divided by the total freshwater produced over the still’s lifetime to give emissions per liter. Operational emissions are negligible unless auxiliary devices (fans or humidifiers) are used, in which case electricity-related CO₂ is added. This yields a grams-of-CO₂-per-liter metric for comparison with fossil-fuel desalination. A pyramidal solar still producing about 0.3 L·m⁻²·day⁻¹ over a 10-year lifespan yields roughly 1080 L per m², with embodied emissions of about 9 g CO₂/L when accounting for both construction and operating conditions. In contrast, grid-powered RO desalination emits around 18 g CO₂/L, considering construction impacts and fossil-fuel energy use. This suggests that the solar still provides measurable CO₂ savings of 9 g CO_2_/L over fossil-based thermal desalination, though its advantage may be limited compared to more efficient RO systems.

### Energy and exergy analysis

The energy analysis is based on the first law of thermodynamics, focusing on the energy input from solar radiation and the useful energy used for water evaporation. Energy efficiency is defined as the ratio of the energy used for evaporation to the energy input from solar radiation. Q_in_ is the energy input in Joules, A is the area of the still basin in m^2^ and t is the time in seconds. Q_evap_ is the energy used for the evaporation in J, m_w_ is the mass of the water in kg and h_fg_ is the latent heat of vaporization, J/kg.

8 $$\:{Q}_{in}=I\text{*}A\text{*}t$$

9 $$\:{Q}_{evap}={m}_{w}\text{*}{h}_{fg}$$

For the time of 5 h, and with the intensity of 1400 W/m^2^, the energy input is obtained as 1.8 × 10^7^ J. Similarly for 2 kg or 2 liters of water used and considering the latent heat of vaporization which is equal to 2260 kJ/kg the value of energy used for evaporation is calculated as 4.52 × 10^6^ J.

10 $$\:{\eta\:}_{energy}=\frac{{Q}_{evap}}{{Q}_{in}}$$

By substituting (8) and (9) in the Eq. (10), the energy efficiency value is determined. It is found to be 25.11%.

Exergy analysis evaluates the quality of energy and identifies the real potential for work within the system. Exergy analysis guides the optimization of design parameters, material selection, and operating conditions to maximize freshwater productivity and improve overall sustainability. Exergy efficiency is defined as a ratio of the exergy used for the evaporation to the exergy of solar radiation. Where $\:{Ex}_{in}$ which is given by Eq. (11) is the exergy used for the solar radiation in J, T_a_ and T_s_ are the ambient and the temperature of the sun which are taken as 313 K and 6000 K respectively. Exergy of solar radiation is found to be 1.7061 × 10^7^ J.

11 $$\:{Ex}_{in}={Q}_{in}\left(1-\frac{{T}_{a}}{{T}_{s}}\right)$$

Exergy associated with the evaporation of the water is given by the Eq. (12). Tw is the water temperature which is considered as 336 K. The exergy associated with evaporation is found to be 3 × 10^5^ J.

12 $$\:{Ex}_{evap}={m}_{w}\text{*}{h}_{fg}\left(1-\frac{{T}_{a}}{{T}_{w}}\right)$$

The exergy efficiency is found using Eq. (13). The value of exergy efficiency is found to be 1.75%. Energy efficiency is significantly higher than exergy efficiency because much of the solar energy is low-quality thermal energy, not fully convertible to useful work. Table 8 gives the values of energy and exergy efficiency and the results are in line with the literature^35^.

13 $$\:{\eta\:}_{exergy}=\frac{{Ex}_{evap}}{{Ex}_{in}}$$

**Table 8:**
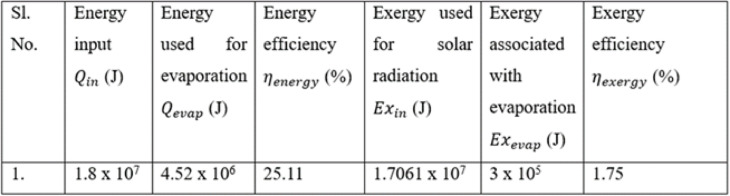
Energy and exergy analysis.

## Conclusions

A pyramidal solar still was developed to compare static, fan-assisted, and ultrasonic humidifier-based configurations, evaluating thermal and distillation performance to assess the effectiveness of passive and active enhancement techniques.

1. The static solar still showed the highest basin, glass, and water temperatures, increasing by 28–31% between 11:00 AM and 3:00 PM due to passive solar heating.
2. The maximum vapor pressures at the basin and glass were 18,626.71 Pa and 15,276.53 Pa, with the ultrasonic humidifier-based still showing 38.04% and 23.87% higher values, respectively.
3. The fan-based still showed a higher condensation coefficient 1.65 W/m²·°C from forced convection, while the ultrasonic still achieved a higher evaporative coefficient 20.99 W/m²·°C by increasing vapor concentration.
4. The ultrasonic humidifier-based still achieved 150.96 W/m² peak heat transfer and 58% efficiency, improving 66.64% and 123.07% over the static still.
5. In a five-hour test 11 AM–4 PM, the ultrasonic still produced 330 ml of distilled water, compared to 180 ml from the dynamic and 90 ml from the static still.
6. The ultrasonic humidifier-based still greatly enhanced heat/mass transfer and distillation efficiency under Dubai’s strong solar radiation.
7. The energy and exergy efficiencies are **25.11%** and **1.75%**, respectively, with exergy lower due to the low-quality thermal nature of solar energy, which is not fully convertible to useful work.

The pyramidal solar still offers a sustainable, low-cost solution to water scarcity by converting abundant solar energy into clean water, reducing fossil fuel dependence and supporting global goals for accessibility and climate resilience.

## Data Availability

Data will be made available by the corresponding author upon request.

## Abbreviations

FRL: Fresnel lens
HHPW: High hourly productivity window
PCM: Phase change material
PV/T: Photovoltaic/thermal
PV: Photovoltaic
ESM: Energy storage materials
PSS: Pyramid solar still
AV: Air Velocity
CPSS: Conventional pyramid solar still
MPSS: Modified pyramid solar still
PWM: Pulse width modulation
DC: Direct current
AC: Alternate current
RH: Relative Humidity
RSS: Root Sum of Squares
HSD: Hemispherical solar distiller
CSD: Conventional solar distiller
SCSBSS: Semi-cylindrical stepped-basin solar still
SCSS: Semi-cylindrical solar still
SSSS: Single slope solar still
CSB: Coconut shell biochar
SSDBSS: Single-slope double-basin solar still
ZZACC: zig-zag air-cooled condenser
RBSD: Rotating spherical solar distiller

## Variables/Parameters

h_ew_: Evaporative heat transfer coefficient
h_cw_: Condensation heat transfer coefficient
P_w_ and P_g_: Saturated vapor pressure at water & glass, Pa
T_W_ and T_g_: Temperature of water and glass, ⁰C
$\:\dot{Q}$: Rate of heat transfer, W
I: Incident radiation, W/m^2^
R: Dependent variable
X: Independent variable
Y: Intervals of uncertainty
G: Function
Y_G_: Total uncertainty
Q_in_: Energy input, Joules
A: Area of the still basin, m^2^
t: Time, seconds
Q_evap_: Energy used for the evaporation, J
m_w_: Mass of the water, kg
h_fg_: Latent heat of vaporization, J/kg
$${\eta\:}_{energy}$$: Energy efficiency, %
T_a_: Ambient temperature, K
T_s_: Sun temperature, K
T_w_: Water temperature, K
${Ex}_{in}$: Exergy of solar radiation, J
$\:{Ex}_{evap}$: The exergy associated with evaporation, J
$$\:{\eta\:}_{exergy}$$: Exergy efficiency, %

## Subscripts

W: Water
g: Glass
G: Uncertainty
R: Overall

## Greek Letters

η: Efficiency, %
$$\:\partial\:\text{and}\triangle$$: Change

## Chemicals

Ni Cr: Nickel Chromium
CuO: Copper Oxide
Al_2_O_3_: Aluminium Oxide
ZnO: Zinc Oxide
C_6_H_5_OH^−^: Phenolic Compounds
Ca^2+^: Calcium
Ca^2+^CO_3_^2−^: Calcium Carbonate
Cl^−^: Chloride
Cl_2_^+^: Residual, free Chlorine, mg/L
F^−^: Fluoride
Mg^2+^: Magnesium
NO_3_^−^: Nitrate
SO_4_^2−^: Sulphate
Cr³⁺: Chromium
Ni²⁺: Nickel
Cu²⁺: Copper
Pb²⁺: Lead
MoS₂: Molybdenum Disulfide
Mo O₃: Molybdenum trioxide
Ni NCs: Nickel nanoclusters
K: Potassium
Ca: Calcium
C_6_H_5_OH^−^: Phenolic Compounds
Ag: Silver
